# Technological advancements in antibody-based therapeutics for treatment of diseases

**DOI:** 10.1186/s12929-025-01190-2

**Published:** 2025-11-12

**Authors:** Ruei-Min Lu, Hsiao-Ling Chiang, Joyce Pei‑Yi Yuan, Hsiu-Hua Wang, Chi-Yung Chen, Sushree Shankar Panda, Kang-Hao Liang, Hung-Pin Peng, Shih-Han Ko, Hung-Ju Hsu, Monika Kumari, Yi-Jen Su, Yi-Ting Tse, Nai-Lin Chou, Han-Chung Wu

**Affiliations:** 1 Biomedical Translation Research Center, Academia Sinica, Taipei, 11571 Taiwan; 2 Institute of Cellular and Organismic Biology, Academia Sinica, Taipei, 11529 Taiwan; 3 Clinical Data Center, Office of Data Science, Taipei Medical University, Taipei, 11031 Taiwan

**Keywords:** Antibody therapeutics, New modalities, Antibody-drug conjugates (ADCs), Bispecific antibodies (bsAbs), Chimeric antigen receptor T (cell therapies CAR-T), Artificial intelligence (AI), Antibody market, Antibody engineering

## Abstract

Monoclonal antibodies (mAbs) represent a major class of therapeutics with widespread clinical applications in oncology, immunology, hematology, neurology and infectious disease. Since the introduction of hybridoma technology in 1975, the field has been advanced by a succession of innovations including chimeric and humanized antibody engineering, phage display, transgenic mouse platforms and high-throughput single B cell isolation. These technological developments have enhanced the specificity, potency and safety of mAbs, resulting in 144 FDA-approved antibody drugs on the market and 1,516 worldwide candidates in clinical development as of August 2025. Engineering breakthroughs have led to new modalities of antibody-based therapeutics, such as antibody-drug conjugates (ADCs), bispecific antibodies (bsAbs), and chimeric antigen receptor T (CAR-T) cell therapies. Each of these modalities has therapeutic utility across multiple disease domains. Recent advances in delivery strategies, notably mRNA-lipid nanoparticles (LNPs) and antibody-directed in vivo CAR-T cell reprogramming, can enable precision therapies while reducing off-target effects and manufacturing complexity. The integration of artificial intelligence (AI) and machine learning (ML), next-generation sequencing (NGS), and structural modeling tools has further accelerated antibody discovery, affinity maturation and immunogenicity prediction, allowing for more efficient and rational antibody design. The advances in antibody technology are reflected in the rapid market growth of antibody-based therapeutics, which had global sales exceeding USD 267 billion in 2024. This review provides a comprehensive update on recent developments in antibody discovery platforms, therapeutic formats and market trends, highlighting emerging strategies that are reshaping the landscape of antibody-based medicine. Furthermore, we discuss clinical translation, regulatory landscapes, and the integration of engineering, biology and informatics. Together, these aspects shape a dynamic and multidisciplinary future for the therapeutic antibody field, which is poised to address unmet clinical needs and global healthcare priorities.

## Introduction

Monoclonal antibodies (mAbs) have revolutionized biomedical research and clinical practice, serving as indispensable tools in biochemistry, molecular/cellular biology, and clinical therapeutics. The application of mAbs as therapeutic agents has expanded dramatically over the past decades, leading to a growing portfolio of antibody-based treatments for cancer, autoimmune disorders, infectious diseases and neurological conditions. The global market for therapeutic antibodies has likewise grown at an extraordinary pace, achieving USD 267 billion in annual sales by 2024 (GlobalData, August 2025). This expansion reflects not only the clinical success of antibody-based therapies but also the diversification of molecular targets and disease indications. Notably, top-selling agents, such as pembrolizumab (Keytruda), dupilumab (Dupixent) and adalimumab (Humira), exemplify the unparalleled position of immuno-oncology and anti-inflammatory biologics within the pharmaceutical landscape.

**Fig. 1:**
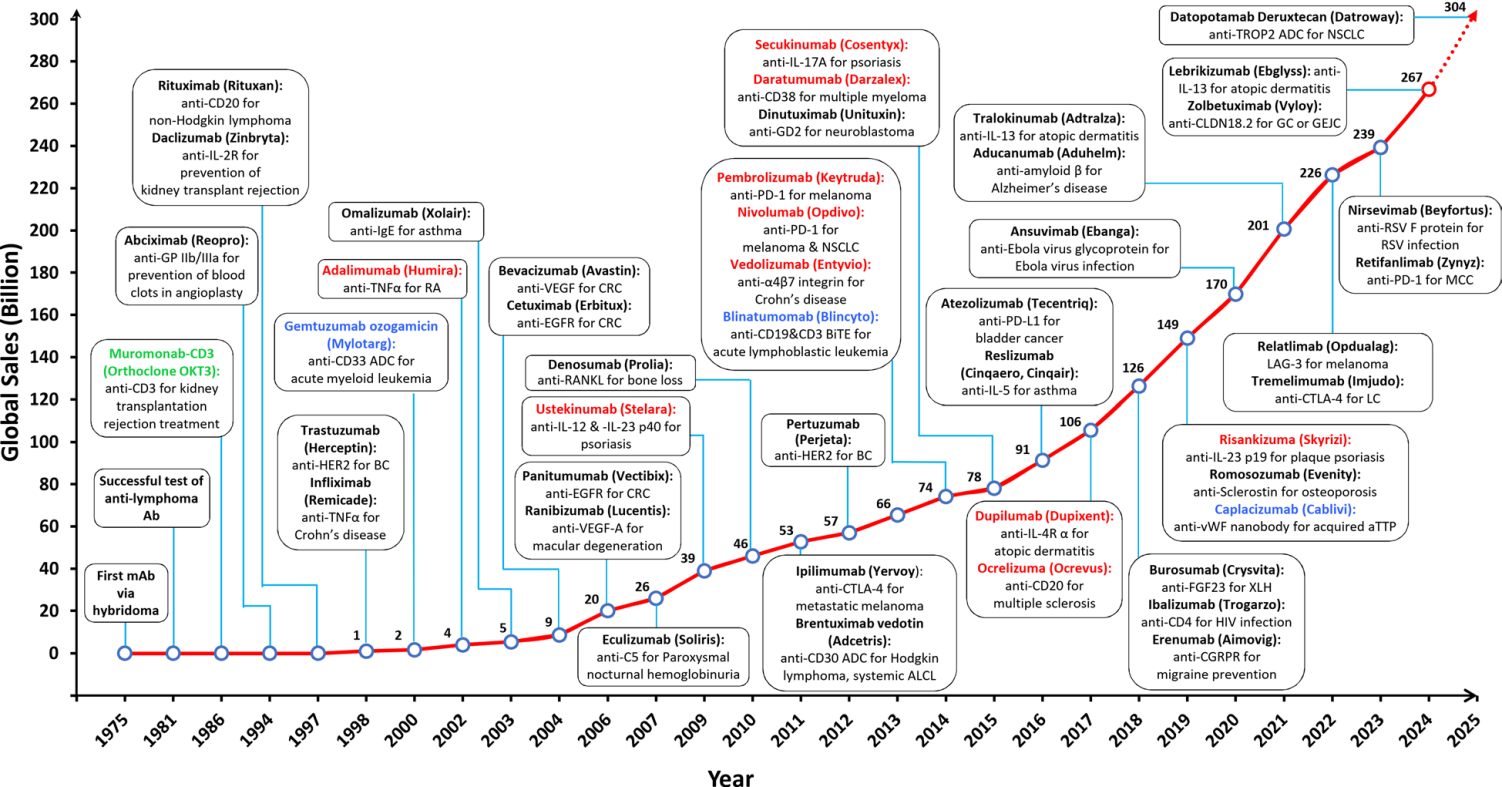
Timeline of therapeutic antibody development and global market growth from 1975 to 2025. The graph illustrates the evolution of therapeutic antibodies, highlighting key milestones in development and the U.S. FDA approvals, alongside the corresponding global market size (in billions USD). Each annotated box represents an FDA-approved antibody, detailing its generic name, trade name and therapeutic indication. Milestones include the introduction of the first mAb in 1975, the first approval mAb in 1986 (green), and subsequent approvals of pivotal antibodies such as rituximab (1997) for non-Hodgkin lymphoma and adalimumab (2002) for rheumatoid arthritis. The market size (depicted by the red line) exhibits a consistent upward trajectory, rising from near zero in 1975 to a projected USD 267 billion in 2024. Antibodies highlighted in bold red text signify the top 10 global sales in 2024, underscoring their significant commercial impacts in oncology, autoimmune diseases, and other therapeutic areas. The first U.S. FDA-approved ADC, bsAb and nanobody are highlighted in bold blue text. ALCL, systematic anaplastic large-cell lymphoma; aTTP, acquired thrombotic thrombocytopenic purpura; BC, breast cancer; CD, cluster of differentiation; CGRPR, calcitonin gene-related peptide receptor; CRC, colorectal cancer; CTLA-4, cytotoxic T-lymphocyte-associated protein 4; EGFR, epidermal growth factor receptor; FGF, fibroblast growth factor; GC, gastric cancer; GD2, disialoganglioside GD2; GEJC, gastroesophageal junction cancer; HER2, human epidermal growth factor receptor 2; IgE, immunoglobulin E; IL, interleukin; IL-17R, interleukin-17 receptor; LAG-3, lymphocyte-activation gene 3; LC, liver cancer; MCC, merkel-cell carcinoma; NSCLC, non-small cell lung cancer; PD-1, programmed cell death protein 1; PD-L1, programmed death-ligand 1; TNFα, tumor necrosis factor α; RA, rheumatoid arthritis; RANKL, receptor activator of nuclear factor kappa-B ligand; VEGF-A, vascular endothelial growth factor A; XLH, X-linked hypophosphatemia

In Fig. 1, we illustrate the chronological evolution of FDA-approved mAbs, highlighting key milestones such as the first anti-CD3 antibody and subsequent innovations of immune checkpoint inhibitors, antibody-drug-conjugates (ADCs) and bispecific antibodies (bsAbs). As of August 2025, a total of 1,516 therapeutic antibody products (excluding biosimilars and combination therapies) are in clinical development worldwide. Sixteen have pre-registration status, and 207 are already marketed globally. Among these marketed mAbs, 144 mAbs have been approved by the FDA and are currently available on the market (GlobalData, August 2025). Table 1 provides a representative overview of these approved therapeutics, detailing the targets, indications and their sponsoring companies. In addition, we highlight the discovery platforms used in development, which include hybridoma, phage display, transgenic models and single B cell technologies. Notably, the integration of molecular design and commercial strategy has allowed the antibody platform to become a central pillar of biopharmaceuticals, as advances in engineering can be used to significantly enhance the specificity, efficacy and safety of different products.

**Table 1:** U.S. FDA-approved monoclonal antibodies on the market

No	Generic name	Brand name	Company	Target	Format	Technology	Indication^&^	U.S. approval
1	Abciximab	Reopro	Centocor/Eli Lilly/Janssen Biotech	GPIIb/IIIa	Chimeric IgG1 Fab	Hybridoma	Prevention of blood clots in angioplasty	1994
2	Rituximab	MabThera, Rituxan	Biogen/Roche/Genentech	CD20	Chimeric IgG1	Hybridoma	Non-Hodgkin lymphoma	1997
3	Basiliximab	Simulect	Bristol Myers Squibb	IL-2R	Chimeric IgG1	Hybridoma	Prevention of kidney transplant rejection	1998
4	Palivizumab	Synagis	MedImmune/AbbVie	RSV F protein	Humanized IgG1	Hybridoma	Prevention of RSV infection	1998
5	Infliximab	Remicade	Janssen Biotech	TNFα	Chimeric IgG1	Hybridoma	Crohn’s disease	1998
6	Trastuzumab	Herceptin	Roche/Genentech	HER2	Humanized IgG1	Hybridoma	Breast cancer	1998
7	Adalimumab	Humira	AbbVie	TNFα	Human IgG1	Phage display	Rheumatoid arthritis	2002
8	Ibritumomab tiuxetan	Zevalin	Biogen/Schering AG/Spectrum Pharmaceuticals	CD20	Murine IgG1	Hybridoma	Non-Hodgkin lymphoma	2002
9	Omalizumab	Xolair	Genentech/Roche, Novartis Pharmaceuticals	IgE	Humanized IgG1	Hybridoma	Asthma	2003
10	Cetuximab	Erbitux	Bristol-Myers Squibb/Merck/Eli Lilly/ImClone Systems	EGFR	Chimeric IgG1	Hybridoma	Colorectal cancer	2004
11	Bevacizumab	Avastin	Roche/Genentech	VEGF-A	Humanized IgG1	Hybridoma	Colorectal cancer	2004
12	Natalizumab	Tysabri	Biogen/Elan Pharmaceuticals International	α4 Integrin	Humanized IgG4	Hybridoma	Multiple sclerosis	2004
13	Panitumumab	Vectibix	Amgen	EGFR	Human IgG2	Transgenic mice/XenoMouse	Colorectal cancer	2006
14	Ranibizumab	Lucentis	Roche/Genentech/Novartis	VEGF-A	Humanized IgG1 Fab	Hybridoma	Macular degeneration	2006
15	Eculizumab	Soliris	Alexion/AstraZeneca	Complement component 5 (C5)	Humanized IgG2/4	Hybridoma	Paroxysmal nocturnal hemoglobinuria	2007
16	Certolizumab pegol	Cimzia	Celltech, UCB	TNFα	Humanized Fab, pegylated	Hybridoma	Crohn’s disease	2008
17	Ustekinumab	Stelara	Medarex/Janssen Biotech	IL-12&IL-23 p40	Human IgG1	Transgenic mice/HuMab Mouse	Psoriasis	2009
18	Canakinumab	Ilaris	Novartis Pharmaceuticals	IL-1β	Human IgG1	Transgenic mice/HuMab Mouse	Muckle-Wells syndrome	2009
19	Golimumab	Simponi	Centocor Ortho Biotech/Janssen Biotech	TNFα	Human IgG1	Transgenic mice/HuMab Mouse	Rheumatoid and psoriatic arthritis, ankylosing spondylitis	2009
20	Ofatumumab	Arzerra	Genmab A/S/GlaxoSmithKline/Novartis	CD20	Human IgG1	Transgenic mice/HuMab Mouse	Chronic lymphocytic leukemia	2009
21	Tocilizumab	RoActemra, Actemra	Chugai Pharmaceutical/Roche/Genentech	IL-6R	Humanized IgG1	Hybridoma	Rheumatoid arthritis	2010
22	Denosumab	Xgeva, Prolia	Amgen	RANKL	Human IgG2	Transgenic mice/XenoMouse	Bone loss	2010
23	Belimumab	Benlysta	GlaxoSmithKline/Human Genome Sciences	BLyS	Human IgG1	Phage display	systemic lupus erythematosus (SLE)	2011
24	Ipilimumab	Yervoy	Bristol-Myers Squibb/Medarex	CTLA-4	Human IgG1	Transgenic mice/HuMab Mouse	Metastatic melanoma	2011
25	Brentuximab vedotin	Adcetris	Seattle genetics/Takeda Pharmaceutical	CD30	Chimeric IgG1; ADC	Hybridoma	Hodgkin lymphoma, systemic anaplastic large cell lymphoma	2011
26	Pertuzumab	Perjeta	Roche/Genentech	HER2	Humanized IgG1	Hybridoma	Breast Cancer	2012
27	Raxibacumab	Abthrax	MedImmune/Emergent BioSolutions	*B. anthrasis PA*	Human IgG1	Phage display	Anthrax infection	2012
28	Trastuzumab emtansine	Kadcyla	Roche/Genentech/ImmunoGen	HER2	Humanized IgG1; ADC	Hybridoma	Breast cancer	2013
29	Obinutuzumab	Gazyva, Gazyvaro	Biogen/Roche/Genentech	CD20	Humanized IgG1 Glycoengineered	Hybridoma	Chronic lymphocytic leukemia	2013
30	Siltuximab	Sylvant	Janssen Biotech	IL-6	Chimeric IgG1	Hybridoma	Castleman disease	2014
31	Ramucirumab	Cyramza	Eli Lilly/ImClone Systems	VEGFR2	Human IgG1	Phage display	Gastric cancer	2014
32	Vedolizumab	Entyvio	Takeda Pharmaceuticals	α4β7 integrin	Humanized IgG1	Hybridoma	Ulcerative colitis, Crohn’s disease	2014
33	Nivolumab	Opdivo	Bristol-Myers Squibb/Ono Pharmaceutical	PD-1	Human IgG4	Transgenic mice/HuMab Mouse	Melanoma, non-small cell lung cancer (NSCLC)	2014
34	Pembrolizumab	Keytruda	Merck & Co	PD-1	Humanized IgG4	Hybridoma	Melanoma	2014
35	Blinatumomab	Blincyto	Amgen	CD19, CD3	Murine bispecific tandem scFv	Hybridoma	Acute lymphoblastic leukemia	2014
36	Alemtuzumab	Lemtrada	Genzyme/Sanofi	CD52	Humanized IgG1	Hybridoma	Multiple sclerosis; Chronic myeloid leukemia^&^	2014 2001^#^
37	Evolocumab	Repatha	Amgen/Amgen Astellas BioPharma K.K	PCSK9	Human IgG2	Transgenic mice/XenoMouse	High cholesterol	2015
38	Idarucizumab	Praxbind	Boehringer Ingelheim Pharmaceuticals	Dabigatran	Humanized Fab	Hybridoma	Reversal of dabigatran-induced anticoagulation	2015
39	Necitumumab	Portrazza	Eli Lilly/ImClone Systems	EGFR	Human IgG1	Phage display	NSCLC	2015
40	Dinutuximab	Unituxin	United Therapeutics Corporation	GD2	Chimeric IgG1	Hybridoma	Neuroblastoma	2015
41	Secukinumab	Cosentyx	Novartis Pharmaceuticals	IL-17A	Human IgG1	Transgenic mice/XenoMouse	Psoriasis	2015
42	Mepolizumab	Nucala	Centocor Inc./GlaxoSmithKline	IL-5	Humanized IgG1	Hybridoma	Severe eosinophilic asthma	2015
43	Alirocumab	Praluent	Regeneron Pharmaceuticals/Sanofi	PCSK9	Human IgG1	Transgenic mice/VelocImmune	High cholesterol	2015
44	Daratumumab	Darzalex	Genmab A/S/Janssen Biotech I	CD38	Human IgG1	Transgenic mice/HuMab Mouse	Multiple myeloma	2015
45	Elotuzumab	Empliciti	Bristol-Myers Squibb/AbbVie	SLAMF7	Humanized IgG1	Hybridoma	Multiple myeloma	2015
46	Ixekizumab	Taltz	Eli Lilly	IL-17A	Humanized IgG4	Phage display (mouse Fab library)	Psoriasis	2016
47	Reslizumab	Cinqaero, Cinqair	Celltech, UCB/Schering-Plough/Teva Pharmaceutical	IL-5	Humanized IgG4	Hybridoma	Asthma	2016
48	Bezlotoxumab	Zinplava	Merck & Co	*Clostridium difficile toxin B*	Human IgG1	Transgenic mice/HuMab Mouse	Prevention of *Clostridium difficile* infection recurrence	2016
49	Atezolizumab	Tecentriq	Roche/Genentech	PD-L1	Humanized IgG1	Phage display	Bladder cancer	2016
50	Obiltoxaximab	Anthim	Elusys Therapeutics	*B. anthrasis PA*	Chimeric IgG1	Hybridoma	Prevention of inhalational anthrax	2016
51	Brodalumab	Siliq, Lumicef	MedImmune/Amgen/Kyowa Hakko Kirin/AstraZeneca/Valeant Pharmaceuticals	IL-17R	Human IgG2	Transgenic mice/XenoMouse	Plaque psoriasis	2017
52	Dupilumab	Dupixent	Regeneron/Sanofi	IL-4Rα	Human IgG4	Transgenic mice/VelocImmune	Atopic dermatitis	2017
53	Inotuzumab ozogamicin	Besponsa	Wyeth Pharmaceuticals/Pfizer	CD22	Humanized IgG4; ADC	Hybridoma	Acute lymphoblastic leukemia	2017
54	Guselkumab	Tremfya	MorphoSys/Janssen Biotech	IL-23 p19	Human IgG1	Phage display	Plaque psoriasis	2017
55	Sarilumab	Kevzara	Regeneron/Sanofi	IL-6R	Human IgG1	Transgenic mice/VelocImmune	Rheumatoid arthritis	2017
56	Avelumab	Bavencio	Merck Serono International/Pfizer	PD-L1	Human IgG1	Phage display	Merkel cell carcinoma	2017
57	Emicizumab	Hemlibra	Chugai Pharmaceutical/Roche	Factor IXa, X	Humanized IgG4, bispecific	Hybridoma	Hemophilia A	2017
58	Ocrelizumab	Ocrevus	Biogen/Roche/Genentech/SIGMA-TAU	CD20	Humanized IgG1	Hybridoma	Multiple sclerosis	2017
59	Benralizumab	Fasenra	MedImmune/Kyowa Hakko Kirin/AstraZeneca	IL-5Rα	Humanized IgG1	Hybridoma	Asthma	2017
60	Durvalumab	Imfinzi	MedImmune/AstraZeneca	PD-L1	Human IgG1	Transgenic mice/XenoMouse	Bladder cancer	2017
61	Gemtuzumab ozogamicin	Mylotarg	Pfizer	CD33	Humanized IgG4; ADC	Hybridoma	Acute myeloid leukemia	2017 2000^#^
62	Erenumab	Aimovig	Novartis	CGRPR	Human IgG2	Transgenic mice/XenoMouse	Migraine prevention	2018
63	Galcanezumab	Emgality	Eli Lilly	CGRP	Humanized IgG4	Hybridoma	Migraine prevention	2018
64	Burosumab	Crysvita	Kyowa Hakko Kirin/Ultragenyx Pharmaceutical	FGF23	Human IgG1	Transgenic mice/KM mouse	X-linked hypophosphatemia	2018
65	Lanadelumab	Takhzyro	Dyax	Plasma kallikrein	Human IgG1	Phage display	Hereditary angioedema	2018
66	Mogamulizumab	Poteligeo	Kyowa Hakko Kirin	CCR4	Humanized IgG1	Hybridoma	Mycosis fungoides or Sézary syndrome	2018
67	Tildrakizumab	Ilumya	Merck & Co./Sun Pharmaceutical	IL-23 p19	Humanized IgG1	Hybridoma	Plaque psoriasis	2018
68	Fremanezumab	Ajovy	Teva Pharmaceutical	CGRP	Humanized IgG2	Hybridoma	Migraine prevention	2018
69	Ravulizumab	Ultomiris	Alexion Pharmaceuticals/AstraZeneca	C5	humanized IgG2/4	Hybridoma	Paroxysmal nocturnal hemoglobinuria	2018
70	Cemiplimab	Libtayo	Regeneron Pharmaceuticals	PD-1	Human IgG4	Transgenic mice/VelocImmune	Cutaneous squamous cell carcinoma	2018
71	Ibalizumab	Trogarzo	Taimed Biologics/Theratechnologies	CD4	Humanized IgG4	Hybridoma	HIV infection	2018
72	Emapalumab	Gamifant	Swedish Orphan Biovitrum (Sobi)	IFNγ	Human IgG1	Phage display	Primary hemophagocytic lymphohistiocytosis	2018
73	Moxetumomab pasudotox	Lumoxiti	MedImmune/AstraZeneca	CD22	Murine IgG1 dsFv immunotoxin	Hybridoma	Hairy cell leukemia	2018
74	Caplacizumab	Cablivi	Ablynx	von Willebrand factor	Humanized Nanobody (VHH)	Phage display/camelid	Acquired thrombotic thrombocytopenic purpura (aTTP)	2019
75	Risankizumab	Skyrizi	Boehringer Ingelheim Pharmaceuticals/AbbVie	IL-23 p19	Humanized IgG1	Hybridoma	Plaque psoriasis	2019
76	Polatuzumab vedotin	Polivy	Roche	CD79β	Humanized IgG1; ADC	Hybridoma	Diffuse large B-cell lymphoma	2019
77	Romosozumab	Evenity	Amgen/UCB	Sclerostin	Humanized IgG2	Hybridoma	Osteoporosis in postmenopausal women at increased risk of fracture	2019
78	Brolucizumab	Beovu	Novatis AG	VEGF-A	Humanized scFv	Hybridoma/rabbit	Neovascular age-related macular degeneration	2019
79	Crizanlizumab	Adakveo	Novatis AG	CD62 (P-selectin)	Humanized IgG2	Hybridoma	Sickle cell disease	2019
80	Enfortumab Vedotin	Padcev	Astellas Pharma	Nectin-4	Human IgG1; ADC	Transgenic mice/XenoMouse	Urothelial cancer	2019
81	Trastuzumab deruxtecan	Enhertu	Daiichi Sankyo/AstraZeneca	HER2	Humanized IgG1; ADC	Hybridoma	HER2 + metastatic breast cancer	2019
82	Isatuximab	Sarclisa	Sanofi	CD38	Chimeric IgG1	Hybridoma	Multiple myeloma	2020
83	Sacituzumab govitecan	TRODELVU	Gilead Sciences	TROP2	Humanized IgG1 ADC	Hybridoma	Triple-negative breast cancer (TNBC)	2020
84	Tafasitamab	Monjuvi, Minjuvi	MorphoSys/Incyte	CD19	Humanized IgG1/2 hybrid	Hybridoma	Diffuse large B-cell lymphoma	2020
85	Satralizumab	Enspryng	Chugai Pharmaceutical/Roche	IL-6R	Humanized IgG2	Hybridoma	Neuromyelitis optica spectrum disorder	2020
86	Eptinezumab	VUEPTI	H. Lundbeck A/S	CGRP	Humanized IgG1	Hybridoma/rabbit	Migraine prevention	2020
87	Inebilizumab	Uplizna	Horizon Therapeutics	CD19	Humanized IgG1	Hybridoma	Neuromyelitis optica spectrum disorders	2020
88	Teprotumumab	Tepezza	Horizon Therapeutics	IGF-1R	Human IgG1	Transgenic mice/HuMab Mouse	Thyroid eye disease	2020
89	Atoltivimab, Maftivimab and odesivimab	Inmazeb	Regeneron	Ebola virus	Human IgG1	Transgenic mice/VelocImmune	Ebola virus infection	2020
90	Naxitamab	DANYELZA	Y-mAbs Therapeutics	GD2	Humanized IgG1	Hybridoma	High-risk neuroblastoma and refractory osteomedullary disease	2020
91	Margetuximab	MARGENZA	MacroGenics	HER2	Chimeric IgG1	Hybridoma	HER2 + metastatic breast cancer	2020
92	Ansuvimab	Ebanga	Ridgeback Biotherapeutics	Ebola virus glycoprotein	Human IgG1	Human B cell	Ebola virus infection	2020
93	Evinacumab	Evkeeza	Regeneron	Angiopoientin-like 3	Human IgG4	Transgenic mice/VelocImmune	Homozygous familial hypercholesterolemia	2021
94	Dostarlimab	Jemerli	GlaxoSmithKline (GSK)	PD-1	Humanized IgG4	Hybridoma	Endometrial cancer	2021
95	Amivantamab	RYBREVANT	Johnson & Johnson	EGFR, c-MET	Human/humanized IgG1 Bispecific	EGFR: Transgenic mice/HuMab Mouse c-MET: Hybridoma	NSCLC w/EGFR exon 20 insertion mutations	2021
96	Tralokinumab	Adtralza	LEO Pharma	IL-13	Human IgG4	Phage display	Atopic dermatitis	2021
97	Anifrolumab	Saphnelo	AstraZeneca; MedImmune	IFNAR1	Human IgG1	Transgenic mice/HuMab Mouse Hybridoma	Systemic lupus erythematosus	2021
98	Loncastuximab tesirine	Zynlonta	ADC Therapeutics	CD19	Chimeric IgG1; ADC	Hybridoma	Diffuse large B-cell lymphoma	2021
99	Aducanumab	ADUHELM	Biogen/Eisai	Amyloid beta	Human IgG1	Human B cell	Alzheimer’s disease	2021
100	Tisotumab vedotin	TIVDAK	Genmab/Seagen	Tissue factor	Human IgG1; ADC	Transgenic mice/HuMab Mouse	Cervical cancer	2021
101	Tezepelumab	Tezspire	Amgen/AstraZeneca	Thymic stromal lymphopoietin	Human IgG2	Transgenic mice/XenoMouse	Severe asthma	2021
102	Faricimab	Vabysmo	Roche/Genentech	VEGF-A, Ang-2	Humanized/humanIgG1 Bispecific	VEGF-A: Hybridoma Ang-2: phage display	Neovascular age-related macular degeneration, diabetic macular edema	2022
103	Sutimlimab	Enjaymo	Sanofi	C1s	Humanized IgG4	Hybridoma	Cold agglutinin disease	2022
104	Spesolimab	SPEVIGO®	Boehringer Ingelheim	IL-36R	Humanized IgG1	Hybridoma	Generalized pustular psoriasis	2022
105	Teplizumab	TZIELD	Provention Bio/anofi	CD3	Humanized IgG1	Hybridoma	Type 1 diabetes	2022
106	Ublituximab	BRIUMVI	TG Therapeutics	CD20	Chimeric IgG1	Hybridoma	Multiple sclerosis	2022
107	Tebentafusp	KIMMTRAK	Immunocore	gp100, CD3	Humanized scFv fused with TCR Bispecific	Hybridoma	Metastatic uveal melanoma	2022
108	Relatlimab	Opdualag(relatlimab + nivolumab combo)	Bristol-Myers Squibb	LAG-3	Human IgG4	Transgenic mice/HuMab Mouse	Melanoma	2022
109	Mosunetuzumab	Lunsumio	Roche/Genentech	CD20, CD3	Humanized IgG1 Bispecific	Hybridoma	Follicular lymphoma	2022
110	Teclistamab	TECVAYLI	Janssen (Johnson & Johnson)	BCMA, CD3	Humanized IgG4 Bispecific	Hybridoma	Multiple myeloma	2022
111	Tremelimumab	Imjudo	AstraZeneca	CTLA-4	Human IgG2	Transgenic mice/XenoMouse	Liver cancer	2022
112	Mirvetuximab soravtansine	ELAHERE	ImmunoGen	FRα	Chimeric IgG1; ADC	Hybridoma	Ovarian cancer	2022
113	Lecanemab	Leqembi	Eisai/Biogen	Amyloid beta protofibrils	Humanized IgG1	Hybridoma	Alzheimer's disease	2023
114	Retifanlimab	Zynyz	Incyte	PD-1	Humanized IgG4	Hybridoma	Merkel cell carcinoma	2023
115	Bimekizumab	Bimzelx	UCB Pharma	IL-17A and IL-17F	Humanized IgG1	Hybridoma	Psoriasis	2023
116	Elranatamab	Elrexfio	Pfizer	BCMA, CD3	Humanized IgG2 Bispecific	Hybridoma	Multiple myeloma	2023
117	Epcoritamab	TEPKINLY, EPKINLY™	Genmab/AbbVie	CD20, CD3	Humanized IgG1 Bispecific	Hybridoma	Diffuse large B-cell lymphoma	2023
118	Glofitamab	COLUMVI®	Roche/Genentech	CD20, CD3e	Humanized; Fab-Fc × Fab-Fab-Fc Bispecific	Hybridoma	Diffuse large B-cell lymphoma	2023
119	Mirikizumab	Omvoh	Eli Lilly	IL-23p19	Humanized IgG4	Hybridoma	Ulcerative colitis	2023
120	Nirsevimab	Beyfortus	AstraZeneca/Sanofi	RSV F protein	Human IgG1	Human B cell	Prevention of RSV infection	2023
121	Pozelimab	VEOPOZ	Regeneron Pharmaceuticals	C5	Human IgG4	Transgenic mice/VelocImmune	CHAPLE disease	2023
122	Rozanolixizumab	RYSTIGGO®	UCB Pharma	FcRn	Humanized IgG4	Hybridoma	Generalized myasthenia gravis	2023
123	Talquetamab	Talvey	Janssen (Johnson & Johnson)	GPCR5D, CD3	Humanized IgG4; Bispecific	Hybridoma	Multiple myeloma	2023
124	Toripalimab	LOQTORZI, Tuoyi	Junshi Biosciences	PD-1	Humanized IgG4	Hybridoma	Nasopharyngeal carcinoma	2023
125	Tislelizumab	Tevimbra	BeiGene	PD-1	Humanized IgG4	Hybridoma	Esophageal squamous cell carcinoma	2024
126	Tarlatamab	IMDELLTRA	Amgen	DLL3, CD3	Human scFvs Bispecific	Transgenic mice/XenoMouse	Small cell lung cancer	2024
127	Donanemab	Kisunla	Eli Lilly	Amyloid beta, N3pG (N-terminal truncated)	Humanized IgG1	Hybridoma	Alzheimer's disease	2024
128	Crovalimab	PiaSky	Roche/Genentech	C5	Humanized IgG1	Hybridoma	Atypical hemolytic uremic syndrome	2024
129	Zolbetuximab	VYLOY	Astellas Pharma	Claudin-18.2	Chimeric IgG1	Hybridoma	HER2-negative gastric or gastroesophageal junction adenocarcinoma	2024
130	Marstacimab	Hympavzi	Pfizer	Tissue factor pathway inhibitor	Human IgG1	Phage display	Hemophilia	2024
131	Axatilimab	Niktimvo	Syndax Pharmaceuticals	CSF-1R	Humanized IgG4	Hybridoma	Graft vs. host disease	2024
132	Nemolizumab	Mitchga, Nemluvio	Chugai Pharmaceutical	IL-31Rα	Humanized IgG2	Hybridoma	Pruritus with atopic dermatitis	2024
133	Zanidatamab	Ziihera	Jazz Pharmaceuticals/Zymeworks	HER2, HER2 (biparatopic)	Humanized scFv-Fc × Fab-Fc Bispecific	Hybridoma	Biliary tract cancers	2024
134	Concizumab	Alhemo	Novo Nordisk	Tissue factor pathway inhibitor	Humanized IgG4	Hybridoma	Hemophilia A or B	2024
135	Cosibelimab	Unloxcyt	Checkpoint Therapeutics	PD-L1	Human IgG1	Phage display	Squamous cell carcinoma	2024
136	Lebrikizumab	Ebglyss	Eli Lilly/Almirall	IL-13	Humanized IgG4	Hybridoma	Atopic dermatitis	2024
137	Zenocutuzumab	Bizengri	Merus N.V	HER2, HER3	Humanized IgG1 Bispecific	Hybridoma	Neuregulin 1 fusion (NRG1+) non-small cell lung (NSCLC) and NRG1 + pancreatic (PDAC) cancer	2024
138	Datopotamab deruxtecan	Datroway	Daiichi Sankyo/AstraZeneca	TROP2	Humanized IgG1; ADC	Hybridoma	NSCLC	2025
139	Penpulimab	ANNIKO	Akeso Biopharma	PD-1	Humanized IgG1	Hybridoma	Nasopharyngeal Carcinoma	2025
140	Nipocalimab	Imaavy	Janssen (Johnson & Johnson)	FcRn p51	Human IgG1	N/A	Generalized myasthenia gravis	2025
141	Telisotuzumab vedotin	Emrelis	AbbVie Inc	c-MET	Humanized IgG1; ADC	Hybridoma	NSCLC	2025
142	Clesrovimab	Enflonsia	Merck & Co. Inc	RSV F protein	Human IgG1	Human B cell	Prevention of RSV infection	2025
143	Garadacimab	Andembry	CSL Behring	Factor XIIa	Human IgG4	Phage display	Hereditary angioedema	2025
144	Linvoseltamab	Lynozyfic	Regeneron Pharmaceuticals	BCMA, CD3	Human IgG4; Bispecific	Transgenic mice/VelocImmune	Multiple myeloma	2025
*Withdrawn by the U.S. FDA*								
1	Muromonab-CD3	Orthoclone OKT3	Centocor Ortho Biotech Products LP	CD3	Murine IgG2a	Hybridoma	Kidney transplant rejection	1986^#^ (2011*)
2	Efalizumab	Raptiva	Genentech/Merck KGaA	CD11a	Humanized IgG1	Hybridoma	Psoriasis	2003^#^ (2009*)
3	Tositumomab-I131	Bexxar	GlaxoSmithKline (GSK)/Corixa Corporation	CD20	Murine IgG2a	Hybridoma	Non-Hodgkin lymphoma	2003^#^ (2013*)
4	Daclizumab	Zinbryta; Zenapax	Roche/Biogen/AbbVie	IL-2R	Humanized IgG1	Hybridoma	Multiple sclerosis; prevention of kidney transplant rejection^&^	2016 (2018*); 1997^#^ (2011*)
5	Olaratumab	Lartruvo	Eli Lilly/ImClone Systems Inc	PDGFRα	Human IgG1	Transgenic mice/HuMab Mouse	Soft tissue sarcoma	2016^#^ (2020*)
6	Belantamab mafodotin	BLENREP	GlaxoSmithKline (GSK)	BCMA	Humanized IgG1 ADC	Hybridoma	Multiple myeloma	2020^#^ (2023*)

^*^Information on the withdrawal date comes from the U.S. FDA

^#^Year of the first U.S. FDA approval

^&^Indication of the first U.S. FDA approval

The field has witnessed transformative innovations since the pioneering work of Köhler and Milstein in 1975, who introduced the hybridoma technique for mAb production [1]. From the early approval of murine mAbs, such as muromonab-CD3 [2], to the development of humanized and fully human antibodies (hAbs), technological breakthroughs have propelled mAbs to the forefront of precision medicine. Advances such as phage display [3], transgenic mice [4, 5], single B-cell sorting [6–8], and mRNA-lipid nanoparticle (LNP) technology [9, 10] have significantly accelerated the discovery and optimization of therapeutic antibodies, facilitating their clinical translation and commercial success.

One of the major challenges in early antibody therapeutic development was the high immunogenicity of murine-derived antibodies. To address this issue, chimeric antibodies (chAbs) were created and helped to mitigate immunogenicity issues. The first chAb, abciximab, was approved by the FDA in 1994 for inhibition of platelet aggregation, as well as and first therapeutic chAb for oncology (rituximab) in 1997 for treatment of non-Hodgkin's lymphoma [11].

The introduction of chAbs was followed by the creation of humanized antibodies via complementary-determining region (CDR) grafting retain specificity while reducing immunogenicity [12, 13]. The approach was used to develop the first approved humanized mAb, daclizumab (approved in 1997), which targets anti-IL-2 receptor for prevention of transplant rejection prevention [14]. Trastuzumab (Herceptin) targets the membrane protein HER2 and was the first humanized mAb approved for use in oncology in 1998 [15].

The next breakthrough in antibody technology was the development of fully hAbs through phage display technology, which was pioneered by George P. Smith in 1985 [3]. Gregory P. Winter later refined the technique by introducing single-chain fragment variable (scFv) formats, facilitating the efficient selection of high-affinity antibodies [16, 17]. Adalimumab (Humira) targeting tumor necrosis factor α (TNFα) was the first hAb developed using phage display to be approved in 2002 [18]. The phage display technique can be used not only to identify scFvs and Fabs, but it can also be applied for nanobody (VHH) discovery. Each of these applications has led to successful isolation of a wide variety of specific binders for different molecules, including proteins, cell-surface glycans and receptors [19]. To date, 16 phage-displayed antibody drugs have received FDA approval.

Another transformative innovation was the use of transgenic mice for hAb production, which were introduced in 1994. These included the HuMab Mouse [5] and XenoMouse [4, 20, 21] platforms, which incorporate human immunoglobulin (Ig) genes to enable the generation of fully hAbs following immunization. The first transgenic mouse-derived hAb drug, panitumumab (targets EGFR), was approved in 2006 for cancer treatment [22]. Additional platforms, including VelocImmune (VI) [23], KM mice [24], and OmniRat [25], have further expanded the portfolio of hAbs, so far yielding 30 hAbs and three bsAbs with FDA approval. Furthermore, isolating B cells from transgenic mice to generate neutralizing hAbs has led to breakthroughs in infectious disease treatments [26–28].

Single B cell antibody screening platforms represent a powerful and rapid method to generate fully human mAbs, particularly for the application of isolating neutralizing antibodies against infectious diseases such as SARS-CoV-2 [27, 29], Human Immunodeficiency Virus (HIV) infection [30, 31] and severe malaria [32]. Furthermore, advanced technologies such as droplet-based microfluidics and optofluidics allow high-throughput pairing of VH and VL transcripts from individual B cells, followed by NGS for antibody reconstruction [33, 34] to greatly accelerated antibody discovery [35].

Our group published a comprehensive review of antibody therapeutics in 2020, which provides an overview of the antibody generation platforms, antibody engineering technologies and market trends [36]. In the current review, a comprehensive update on the advances in antibody development methods and market values is given. Beyond traditional mAbs, the field has expanded into new modalities with enhanced functionalities, involving ADCs, bsAbs, CAR-T therapies and mRNA-LNP delivery [10].

ADCs represent a major breakthrough technology to selectively eliminate cancer cells via the combination of specific targeting from antibodies and cytotoxic potency of small-molecule drugs. Recent approvals of ADCs targeting HER2, TROP2, and other oncogenic markers underscore the clinical impact of this modality [37, 38]. Meanwhile, bsAbs have emerged as powerful tools for redirecting immune cells to attack tumors, as demonstrated by the success of blinatumomab and emicizumab [39, 40]. Another revolutionary approach is CAR-T therapy, which leverages engineered T cells expressing antibody-derived receptors to recognize and destroy malignant cells. Since the first FDA approval of a CAR-T therapy (tisagenlecleucel) for treatment of hematological cancers in 2017 [41], applications of this modality have expanded toward treating solid tumors and autoimmune diseases [42–45]. BsAbs are engineered to bind two distinct antigens simultaneously, enabling multiple functional effects. This dual-binding capability is particularly useful in cancer immunotherapy, where bsAbs can redirect immune cells, such as T cells, to tumor sites.

The development of mAb therapeutics is being rapidly transformed by advances in ML and AI. ML models have been applied to enhance key antibody properties (e.g., stability and binding affinity) and for immunogenicity prediction, enabling more efficient screening and optimization of candidate antibodies [46–48]. These data-driven approaches improve the linkages between sequence, structure and function, streamlining the discovery process. Recent breakthroughs in AI, particularly structure-prediction tools like AlphaFold-Multimer and AlphaFold 3 [49, 50], have significantly advanced our ability to model antibody-antigen complexes with atomic-level accuracy. In parallel, generative models such as RoseTTAFold and RFdiffusion [51, 52] have enabled de novo design of antibody scaffolds and binding interfaces. These innovations offer powerful new strategies for accelerating antibody development in oncology, infectious disease and beyond.

During the COVID-19 pandemic, mRNA-LNP vaccines, such as BNT162b2 and mRNA-1273, delivered SARS-CoV-2 spike protein mRNAs to elicit robust neutralizing antibody responses, effectively preventing infection and halting viral transmission [53, 54]. Their rapid design, scalable production and remarkable clinical efficacy clearly demonstrated the transformative potential of mRNA-LNP technology [10, 55]. Moreover, mRNA-LNPs have been employed to deliver mRNA encoding mAbs or bsAbs in vivo, resulting in the in situ production of functional mAbs targeting TNF-α and SARS-CoV-2 RBD, as well as bsAbs targeting B7-H3 × CD3 and claudin 6 × CD3 [56–59]. This in situ expression strategy offers several advantages, including extended antibody half-life and the ability to bypass traditional manufacturing pipelines, thus accelerating drug development timelines and reducing production costs [9].

The combination of these cutting-edge approaches is expected to drive the next wave of antibody-based treatments. As the antibody landscape continues to evolve, these technologies will expediate the development of novel biologics that offer improved efficacy, reduced toxicity and broader applicability. This review aims to provide a comprehensive update on the latest advancements in antibody engineering, therapeutic applications and market trends, highlighting innovations that are shaping the future of antibody-based medicine.

## Market and intellectual property

### Market trends for antibody drugs

In 2024, mAb therapies dominated the pharmaceutical market, with six mAbs (pembrolizumab, dupilumab, risankizumab, daratumumab, ustekinumab and nivolumab) out of the world’s top ten best-selling mono drugs falling into this category. Peptide drugs (semaglutide and tirzepatide) held two spots in the top ten, while only one small molecule drug (apixaban) and one human IgG1 Fc Fusion protein (aflibercep) made the list (Table 2a). This distribution reflects a significant shift in drug development priorities, emphasizing the growing importance and commercial success of biologics, particularly mAbs. The rise of antibody-based drugs is largely due to their high specificity, which allows for precise targeting of disease mechanisms while minimizing off-target effects. This feature makes antibodies especially valuable in the treatment of complex conditions, such as cancer, autoimmune disease and chronic inflammatory disorders.

**Table 2a:** Top 10 best-selling mono drugs in 2024

Rank	Drug	Target	Drug type	1st U.S. FDA approval year and indication	Disease type	Company	Revenue in billion (USD)			
							2024	2023	2022	2021
1	Pembrolizumab (Keytruda)	PD-1	Humanized Ab	2014 Melanoma	Cancer	Merck & Co	29.48	25.01	20.94	17.19
2	Semaglutide (Ozempic)	GLP-1	Peptide	2017 Type 2 diabetes mellitus	Metabolic disease	Novo Nordisk	18.42	14.65	9.14	5.17
3	Dupilumab (Dupixent)	IL-4Rα	Human Ab	2017 Eczema	Dermatologic disease	Sanofi, Regeneron	14.15	11.59	8.68	6.2
4	Apixaban (Eliquis)	Factor Xa	Small molecule	2012 Non-Valvular Atrial Fibrillation	Cardiovascular disease	Bristol-Myers Squibb, Pfizer	13.33	12.21	11.79	11.76
5	Risankizumab (Skyrizi)	IL-23 p19	Humanized Ab	2019 Plaque psoriasis	Autoimmune disease	AbbVie	11.72	7.76	5.17	2.94
6	Daratumumab (Darzalex)	CD38	Human Ab	2015 Multiple myeloma	Cancer	Johnson & Johnson	11.67	9.74	7.98	6.02
7	Tirzepatide (Mounjaro)	GIP/GLP-1	Peptide	2022 Type 2 diabetes mellitus	Metabolic disease	Eli Lilly	11.54	5.16	0.48	-
8	Ustekinumab (Stelara)	IL-12 & IL-23 p40	Human Ab	2009 Psoriasis	Autoimmune disease	Johnson & Johnson	10.36	10.86	9.72	9.13
9	Aflibercept (Eylea)	VEGF	hIgG1 Fc fusion protein	2011 Wet Age-Related Macular Degenerations	Ophthalmologic disease	Regeneron	9.55	9.38	9.65	9.24
10	Nivolumab (Opdivo)	PD-1	Human Ab	2014 Melanoma	Cancer	Bristol-Myers Squibb	9.30	9.01	8.25	7.52

The continued commercial success of mAbs also reflects major technological advancements in antibody engineering, bispecific formats and ADCs, which have expanded the therapeutic potential of this class. As scientific understanding of disease biology deepens and demand for precision medicine increases, mAbs are expected to play an even greater role in shaping the future of healthcare. The clinical versatility and robust market performance of this class makes it a cornerstone of modern therapeutics and a key focus for ongoing pharmaceutical innovation.

The mAb market continues to expand steadily, with oncology and autoimmune diseases dominating global revenues in 2024. Pembrolizumab (Keytruda) not only remains the world’s top-selling mAb, but is also the world’s best-selling drug [60] with revenues of USD 29.48 billion (Tables 2a, 2b). Its leading position is supported by a broad and evolving set of oncology indications, including multiple tumor types and increasing use in first-line combination therapies. Dupilumab (Dupixent) ranks second among antibody-based therapies with USD 14.15 billion in revenues, reflecting sustained clinical usage across dermatologic, allergic and respiratory indications. The 2024 approval for its use to treat chronic obstructive pulmonary disease (COPD) may further solidify its role in treating chronic inflammatory diseases (Table 2b).

**Table 2b:** Top 10 best-selling antibody drugs in 2024

Rank	Ab name	Target	U.S. FDA approval year and indications	Disease types	Company	Revenue in billion (USD)			
						2024	2023	2022	2021
1	Pembrolizumab (Keytruda)	PD-1	2014 Melanoma 2015 NSCLC 2016 Head and neck squamous cell carcinoma 2017 Classical Hodgkin lymphoma; Non-squamous NSCLC, irrespective of PD-L1 expression; Urothelial carcinoma; Solid tumor with specific genetic feature; Gastric or gastroesophageal junction cancer expressing PD-L1 2018 Cervical cancer expressing PD-L1; Primary mediastinal large B-cell lymphoma; Non-squamous NSCLC with no EGFR or ALK genomic tumor aberrations, Squamous NSCLC; Hepatocellular carcinoma; Merkel cell carcinoma 2019 Renal cell carcinoma; Squamous cell carcinoma of esophagus; Endometrial carcinoma (certain types) 2020 Bladder cancer, second biomarker-based tumor types; Cutaneous squamous cell carcinoma; Microsatellite instability-high (MSI-H) or mismatch repair deficient (dMMR) colorectal cancer; Triple‑negative breast cancer expressing PD-L1 2021 Esophageal or gastroesophageal junction (GEJ) carcinoma; HER2-positive gastric or GEJ adenocarcinoma 2022 MSI‑H/dMMR advanced endometrial carcinoma 2023 MSI-H or dMMR solid tumors; Urothelial cancer; Biliary tract cancer; HER2-negative gastric or GEJ adenocarcinoma 2024 Cervical cancer; Endometrial carcinoma; Malignant pleural mesothelioma	Cancer	Merck & Co	29.48	25.01	20.94	17.19
2	Dupilumab (Dupixent)	IL-4Rα	2017 Eczema 2018 Asthma 2019 Atopic dermatitis; Chronic rhinosinusitis with nasal polyposis 2022 Eosinophilic esophagitis; Prurigo nodularis 2024 Chronic rhinosinusitis with nasal polyps; Chronic obstructive pulmonary disease (COPD)	Dermatologic disease Allergic disease Respiratory disease	Sanofi, Regeneron	14.15	11.59	8.68	6.2
3	Risankizumab (Skyrizi)	IL-23 p19	2019 Plaque psoriasis 2022 Psoriatic arthritis; Crohn's disease 2024 Ulcerative colitis	Autoimmune disease	AbbVie	11.72	7.76	5.17	2.94
4	Daratumumab (Darzalex)	CD38	2015 Multiple myeloma	Cancer	Johnson & Johnson	11.67	9.74	7.98	6.02
5	Ustekinumab (Stelara)	IL-12 & IL-23 p40	2009 Psoriasis 2013 Psoriatic arthritis 2016 Crohn's disease 2017 Plaque psoriasis 2019 Ulcerative colitis	Autoimmune disease	Johnson & Johnson	10.36	10.86	9.72	9.13
6	Nivolumab (Opdivo)	PD-1	2014 Melanoma 2015 Non-small cell lung cancer; Renal cell carcinoma 2016 Hodgkin lymphoma; Head and neck cancer 2017 Urothelial carcinoma; MSI-H or dMMR colorectal cancer; Hepatocellular carcinoma 2018 Small cell lung cancer 2020 Esophageal squamous cell carcinoma; Pleural mesothelioma 2021 Gastric cancer; gastroesophageal junction cancer, and esophageal adenocarcinoma regardless of PD-L1 expression; Urothelial carcinoma	Cancer	Bristol-Myers Squibb	9.30	9.01	8.25	7.52
7	Adalimumab (Humira)	TNFα	2002 Rheumatoid arthritis 2005 Psoriatic arthritis 2006 Ankylosing spondylitis 2007 Crohn's disease 2008 Chronic plaque psoriasis; Polyarticular juvenile idiopathic arthritis 2012 Ulcerative colitis 2015 Hidradenitis suppurativa 2016 Non-infectious intermediate, posterior and panuveitis 2017 Fingernail psoriasis	Autoimmune disease Inflammatory disease	AbbVie	8.99	14.40	21.24	20.7
8	Ocrelizumab (Ocrevus)	CD20	2017 Multiple sclerosis	Autoimmune disease	Roche	7.51	7.11	6.72	5.63
9	Vedolizumab (Entyvio)	α4β7 integrin	2014 Ulcerative colitis, Crohn's disease	Inflammatory disease	Takeda	6.32	5.54	5.19	4.65
10	Secukinumab (Cosentyx)	IL-17A	2015 Plaque psoriasis 2016 Ankylosing spondylitis, Psoriatic arthritis 2020 Non-radiographic axial spondyloarthritis 2021 Enthesitis-related arthritis 2023 Rheumatic diseases, Hidradenitis suppurativa	Autoimmune disease Inflammatory disease	Novartis	6.14	4.98	4.79	4.72

Sales of autoimmune therapies such as risankizumab (Skyrizi) and ustekinumab (Stelara) continue to grow due to expanded approvals for ulcerative colitis and Crohn’s disease. Meanwhile, daratumumab (Darzalex) and nivolumab (Opdivo) have respectively maintained consistent usage for hematologic and solid tumors. A major shift in market position has been observed for adalimumab (Humira). Once the world’s best seller (in 2021 and 2022), adalimumab has fallen to 7th place with revenues of USD 8.99 billion in 2024, down from over USD 21 billion in 2022. The steep decline is due to loss of market exclusivity and the launch of multiple biosimilars in the U.S. starting in 2023. Ocrelizumab (Ocrevus) for multiple sclerosis, vedolizumab (Entyvio) for inflammatory bowel disease, and secukinumab (Cosentyx) for rheumatic and dermatologic conditions round out the top 10, highlighting the continued growth of antibody therapies for indications beyond oncology (Table 2b).

From 2025 to 2034, the global antibody drug market is projected to grow from USD 304 billion (GlobalData, August 2025) to USD 973.6 billion, corresponding to a compound annual growth rate (CAGR) of 13.81% [61]. Success in the mAb market is largely influenced by indication breadth, combination treatment strategies, and therapeutic area expansion. Meanwhile, the entry of biosimilars is reshaping the landscape for legacy products.

### Clinical applications of antibody drugs

**Fig. 2:**
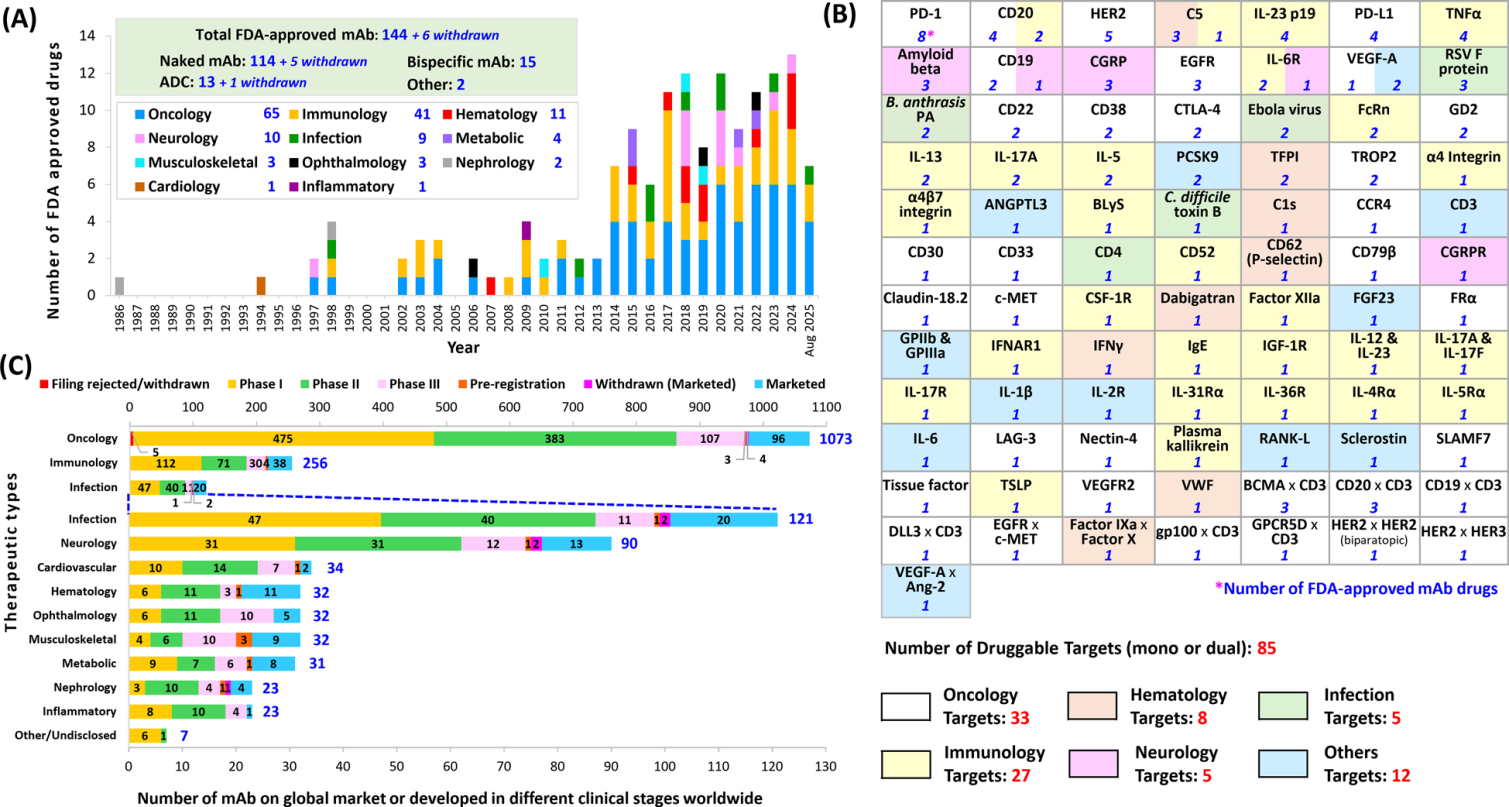
Therapeutic areas and druggable targets of FDA-approved mAbs. **A** Therapeutic areas of FDA-approved antibodies from 1986 to August 2025. There have been 150 mAbs approved by FDA (excluding biosimilars), of which 6 had been withdrawn. Among 150 mAbs, 119 are naked mAbs, 15 are bsAbs, 14 are ADCs, and 2 others are pegylated and immunotoxin-conjugated mAbs. Immunology includes dermatological diseases (psoriasis and atopic dermatitis), gastrointestinal diseases (Crohn’s disease and ulcerative colitis), neurological diseases (multiple sclerosis), respiratory diseases (asthma), rheumatological diseases (rheumatoid arthritis and ankylosing spondylitis), and other immunological diseases (systemic lupus erythematosus, Castleman disease, Chaple disease and graft vs. host disease). Hematology includes paroxysmal nocturnal hemoglobinuria, primary hemophagocytic lymphohistiocytosis, sickle cell disease, hemophilia A or B. Neurology includes migraine prevention, neuromyelitis optical spectrum disorder, Alzheimer's disease. Infection includes RSV, anthrax, Clostridium difficile, HIV, Ebola virus. Metabolic diseases include high cholesterol, homozygous familial hypercholesterolemia, type 1 diabetes. Musculoskeletal disorders include bone loss, X-linked hypophosphatemia, osteoporosis in postmenopausal women. Ophthalmology includes macular degeneration. Nephrology includes prevention of kidney transplant rejection. Cardiology includes prevention of blood clots in angioplasty. Inflammatory includes Muckle-Wells syndrome. **B** Druggable targets and therapeutic areas for 144 FDA-approved mAbs on the market (as listed in Table 1). Therapeutic areas are categorized as oncology, immunology (includes dermatological, gastrointestinal, respiratory, neurological, and rheumatological diseases), hematology, neurology, infection, and others, which include metabolic, musculoskeletal, ophthalmology, nephrology, inflammatory, and cardiology diseases. **C** The 1,754 globally marketed or clinically developed therapeutic mAb innovator entries (not including combination or biosimilars) listed in GlobalData as of August 2025. Among them, 207 mAbs have been approved for market by the FDA and/or other international regulatory agencies, 16 in pre-registration status, 204 in phase III clinical trials, 595 in phase II, 717 in phase I. Additionally, 9 previously marketed mAbs have been withdrawn, and 6 submissions were either rejected or withdrawn

From 1986 to 2025, FDA approvals of therapeutic antibodies have grown significantly, with a notable acceleration starting from 2014 (Fig. 2A). Among therapeutic areas, oncology (44%) is the most common, largely driven by the success of immune checkpoint inhibitors such as anti-PD-1 antibodies (e.g., pembrolizumab and nivolumab, approved in 2014). Immunology (28%) also shows a positive trajectory, particularly regarding the treatment of autoimmune and inflammatory diseases, such as rheumatoid arthritis, psoriasis, asthma, atopic dermatitis and multiple sclerosis. Dupilumab targets IL-4Rα and blocks IL-4 and IL-13 signaling, two key drivers of type 2 inflammation. This drug exemplifies the strategy of growth through multi-indication expansion. After an initial indication of eczema, the drug usage was expanded to asthma, chronic rhinosinusitis with nasal polyposis, eosinophilic esophagitis, prurigo nodularis [62], and COPD. Other areas such as hematology, neurology, infectious disease and metabolic disease have experienced steady growth, while musculoskeletal, nephrology and ophthalmology are emerging as promising fields. Based on these market data, it is clear that the widespread therapeutic versatility and target specificity of antibody-based therapies enables effective and safer treatment options across a wide range of chronic and complex diseases. Overall, these trends reflect the expansion of antibody applications beyond oncology, driven by the capacity of antibody-based drugs to modulate disease pathways with precision and efficacy.

Analysis of FDA-approved antibody targets (Fig. 2B) reveals that PD-1, HER2, CD20, PD-L1 and EGFR are the most frequent targets for approved usages, aligning closely with the fact that oncology is the dominant therapeutic area for antibody drugs. In immunology, key cytokine and receptor targets, such as IL-23 p19, TNFα, IL-5, IL-6R, IL-13 and IL-17A, are commonly targeted for treatment of chronic inflammatory diseases. In neurology, key antibody targets include CGRP for migraine and amyloid-β for Alzheimer’s disease. Antibodies against these targets are intended to modulate key pathological pathways with minimal off-target effects, offering precision treatment for complex brain disorders. Beyond these examples, antibody therapies have been successfully developed for other medical areas, such as anti-PCSK9 for cardiometabolic disease, anti-C5 complement for rare diseases, and antibodies against viral proteins (respiratory syncytial virus [RSV], Ebola virus) or bacterial proteins (*B. anthrasis, C. difficile*) for infectious disease.

An analysis of 1,754 therapeutic mAb entries (excluding combination products and biosimilars) listed in GlobalData (August 2025) revealed that 207 mAbs have been marketed globally, including 144 that are approved by the FDA. Furthermore, 16 mAbs have pre-registration status; 204 are in phase III clinical trials; 595 are in phase II trials; and 717 are in phase I trials. Nine marketed mAbs have been withdrawn from the market, and six regulatory filings have been either rejected or withdrawn (Fig. 2C).

Among the approved or clinical-stage mAb innovators, oncology indications remain the most common applications, accounting for 1,073 (over 61%) mAbs. Immunology indications are listed for 256 candidates, making it the second-largest area (over 15%), with steady development across different autoimmune and inflammatory conditions. Infectious diseases and neurology represent emerging but still underrepresented therapeutic areas, with 121 and 90 mAbs respectively. Hematology, ophthalmology, cardiovascular disease, and metabolic disease are much smaller but gradually expanding fields. These areas comprise many unmet clinical needs and opportunities for innovation. Among 1,516 clinically developed mAbs, 87% are in phase I and II trials, highlighting the fact that target biology and early planning for regulatory alignment, clinical endpoints and patient needs will be essential for successful advancement.

Another notable trend in antibody-based therapeutics is the rise of ADCs. Currently, there are 322 ADC drug entries. A total of 318 are for oncology indications, with 19 are on market worldwide (including 13 FDA approvals on market), 1 in pre-registration status, 40 in phase III trials, 104 in phase II trials, 154 in phase I trials, 1 marketed withdrawn, and 3 filings rejected or withdrawn. The advancement of these drugs underscores the growing maturity and clinical relevance of ADCs, as their targeted cytotoxicity and potential to overcome resistance mechanisms make them especially attractive for difficult-to-treat cancers.

BsAbs are gaining significant momentum in the global biopharmaceutical field, with 20 entries currently marketed and 178 in clinical development worldwide. Among these bsAbs, 148 have primary indications in oncology, reflecting strong clinical and commercial interest in immune cell-redirecting therapies. In addition, 14 candidates are being developed for immunology, 5 for ophthalmologic conditions, 4 for hematologic disorders, 3 for infectious diseases, 3 for musculoskeletal indications and 1 for inflammatory diseases. This profile of indications highlights the growing potential of dual-targeting bsAbs to address multifactorial and underserved diseases beyond cancer.

### Intellectual properties covering antibody drugs

Based on an analysis of 62,114 mAb-related patent entries available in the Cortellis Drug Discovery Intelligence database (Clarivate PLC; August 2025), several major trends can be identified regarding the trajectories of approved antibody drugs in the global market.

#### Geographic distribution of patent filings

The United States leads other countries in patent filings by far, with more 57% of the total filings for mAb-related patents. This high level of activity underscores the central position of the U.S. in biopharmaceutical innovation and suggests an aggressive stance on intellectual property protection in antibody drug development. The European Patent Office (EPO) has 9.5%, while Japan and China follow with 9.0%. Notably, activity in China has shown exponential growth in recent years. United Kingdom, South Korea, and Australia account for 6.0, 4.4, and 1.2% of patent filings. This global spread of activity reflects strategic intellectual property and market positioning in these countries.

#### Indications of interest

About 2,400 indications are addressed by antibody-related patents. Oncology is the most represented therapeutic area, with 48% of total patents focused on this general area. In particular, breast cancer, lung cancer, ovarian cancer, melanoma, pancreatic cancer, prostate cancer, colon cancer, stomach cancer, multiple myeloma, and hematologic malignancies occupy the top 10 oncology indications for different patents. This high level of coverage mirrors the global dominance of cancer immunotherapies (e.g., pembrolizumab and nivolumab) in clinical development and market success. Autoimmune and inflammatory diseases are another major area of interest, with indications including rheumatoid arthritis, multiple sclerosis, asthma, psoriasis and Crohn’s disease, among others. Infectious diseases such as viral and bacterial infection are also notable indications in light of the recent COVID-19 pandemic and emergence of other infectious diseases. Although neurological disorders constitute a smaller share of patent filings, interest in Alzheimer’s and other neurodegenerative diseases is rising steadily, consistent with recent FDA approvals of lecanemab and donanemab.

#### Target-based mechanisms of action

More than 5,000 proteins are targeted by mAbs covered in filed patents. The top 10 most frequently explored target-based actions include PD-1 inhibitors, PD-L1 inhibitors, HER2 inhibitors, CTLA-4 inhibitors, CD3 antagonists, EGFR antagonists, CD20 inhibitors, Fc receptor agonists, CD3 modulators, and TNF-α inhibitors. Patents on antibodies targeting PD-1/PD-L1 exceed 7,000, reflecting a major sustained interest in immune checkpoint inhibition. There is also growing attention paid to CD3-based bsAbs, which enable T cell engagement. This activity is aligned with the trend of bsAb approvals, such as blinatumomab (CD3 × CD19), teclistamab (CD3 × BCMA), talquetamab (CD3 × GPRC5D), and glofitamab (CD3 × CD20).

#### Technology types

Although naked mAbs still dominate the patent landscape, new formats like ADCs, BsAbs and multi-specific antibodies are gaining attraction. The applicability of these technologies has been demonstrated by approvals of ADCs (e.g., trastuzumab emtansine in 2013, trastuzumab deruxtecan in 2019, and sacituzumab govitecan in 2020), the first BiTE (blinatumomab in 2014), and the first nanobody (caplacizumab in 2019).

## Generation and development of mAbs

### mAbs from hybridoma technology

**Fig. 3:**
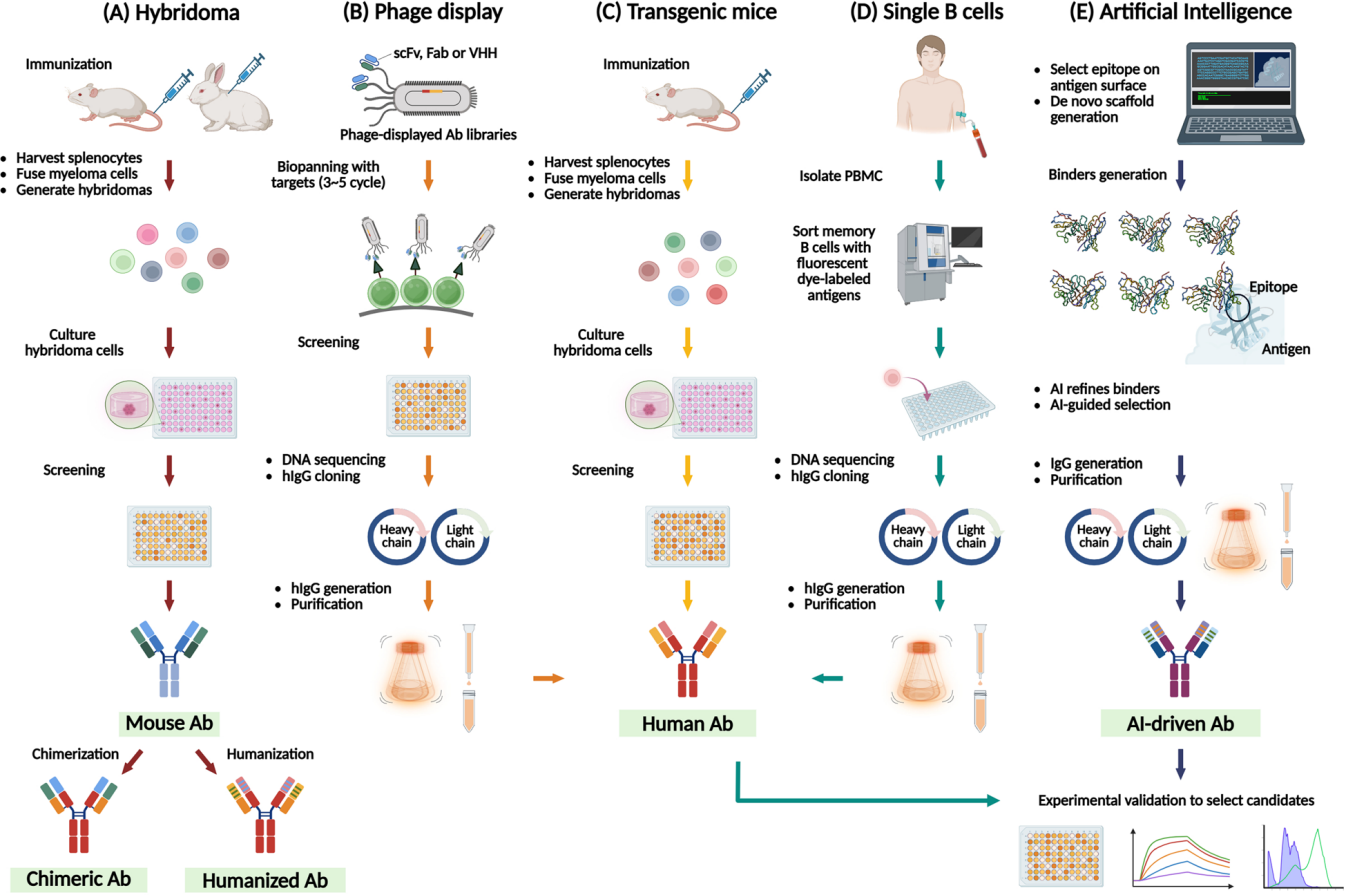
Overview of current methods for generation of therapeutic antibodies. **A** Hybridoma technology: Mice or rabbit are immunized with antigens, followed by harvesting splenocytes and generating hybridomas through fusion with myeloma cells. Hybridoma cells are cultured and screened for antigen-specific clones and cultured to produce mouse antibodies. The VH/VL sequences are identified from hybridoma cells and further engineered into chimeric or humanized antibodies through heavy and light chain replacements. **B** Phage display: scFv, Fab fragments or VHH regions are presented on phage display libraries. Through 3-5 rounds of biopanning, antigen-specific binders are selectively enriched, screened and sequenced for VH and VL regions. These genes are cloned into human IgG/kappa backbone expression vectors and transfected into mammalian cells to produce human antibodies. **C** Transgenic mice: Transgenic mice expressing human antibody genes are immunized, and splenocytes are harvested to generate hybridomas. Hybridoma cells are cultured and screened for antigen-specific clones to produce hAbs. **D** Single B cell technology: PBMCs are isolated from immunized humans, and memory B cells expressing membrane-bound antibodies are sorted using fluorescent dye-labeled antigens and CD markers by FACS. The isolated B cells are used for single-cell RT-PCR to amplify VH/VL genes, followed by DNA sequencing, IgG construction and purification to produce hAbs. Binding affinity of the produced antibodies is validated using ELISA, flow cytometry, and surface plasmon resonance. **E** AI-assisted antibody design: The pipeline leverages AI generative models to design antibody binders targeting a defined epitope. These models generate candidate antibody sequences and structures based on their learned representations of protein architecture and molecular interactions, aiming to complement the epitope’s physicochemical properties as well as the conformational features of the antibody scaffold. The resulting candidates are subsequently assessed by predictive AI models for key attributes, including binding affinity, solubility, thermostability, and immunogenicity. Top-ranked candidates, selected through in silico screening, are then synthesized, cloned into an IgG backbone, and expressed for downstream experimental validation

As one of the most common approaches for mAb production, hybridoma technology is a well-established and indispensable tool for generating high-quality candidates. The process of producing hybridoma mAbs comprise of several key steps, including selecting the antigens, immunizing the host, fusing the cells, cloning and screening, and characterizing the resulting antibodies (Fig. 3A) [63]. In addition to ordinary mouse hybridomas, scientists have utilized CRISPR-Cas9 technology to simplify the hybridoma process by regulating and optimizing the fusion of B cells and myeloma cells. These improvements have led to better antibody screening and enhanced production of antibodies targeting specific antigens [64]. Muromonab-CD3, also known as Orthoclone OKT3, is a purified, mouse-derived mAb that binds to the CD3 antigen on all mature human T cells and was the first mAb approved by the FDA in 1986 [2]. Moreover, the development of hybridoma technology laid the foundation for transgenic mouse platforms. These platforms include the HuMAb Mouse, KM Mouse and VelocImmune. For further details, please refer to Sect. "Antibodies from human Ig transgenic animals".

While mouse-derived hybridomas have long been the cornerstone of therapeutic antibody development, species such as rabbits have also played important roles. Rabbits are particularly well-suited for generating high-affinity research reagents and diagnostic antibodies, owing to their unique immune repertoires and distinct epitope recognition capabilities. Rabbits currently rank second only to mice as hosts for hybridoma technology, with mice remaining the predominant species for generating hybridoma-derived mAbs. In 1995, Katherine Knight and colleagues established the first rabbit plasmacytoma cell line, designated 240E-1, which served as a fusion partner for rabbit B cells [65]. This cell line harbored two oncogenic transgenes, c-myc and v-abl, and exhibited sensitivity to hypoxanthine-aminopterin-thymidine (HAT) selection medium. Notably, 240E-1 could successfully fuse with splenocytes from immunized rabbits and yield stable hybridoma clones. Through iterative subcloning and molecular optimization, an improved derivative cell line, 240E-W, was developed [66]. This advanced fusion partner demonstrated enhanced fusion efficiency and supported more robust and high-quality mAb production.

It is worth noting that rabbits are capable of producing immune-mediated reactions to human antigenic epitopes that particular rodent species cannot identify [67]. The third CDR of the heavy chain (CDR-H3) are longer in rabbits than in mice. These regions are more similar those of human CDR-H3 and enable the rabbits acquire antigenic epitopes [67, 68]. Specifically, rabbits are a valuable source of high-affinity and high-specificity antibodies due to their diverse B cell population, robust immune response, rich VH gene pool, effective affinity maturation process, long lifespan, and potential for self-renewal [69].

#### Humanization of mAbs

Most therapeutic antibodies originate from animals but are modified into humanized forms to reduce immunogenicity. Non-human antibodies can trigger immune responses, such as human anti-mouse antibodies (HAMAs), which decrease efficacy and may cause severe side effects, exemplified by cytokine release syndrome from OKT3 [70–72]. Humanization preserves antigen specificity while minimizing immunogenicity, using strategies such as chimerization, resurfacing, CDR grafting, and specificity-determining residue (SDR) grafting [36, 73]. The first chimeric antibody, abciximab, approved in 1994 for ischemic heart disease, marked the success of this approach.

The most widely used approach of humanization is CDR grafting, in which CDRs from a non-human antibody are transferred onto a human antibody framework [12]. To further refine humanization outcomes, multiple framework selection strategies have been introduced. Framework-homology-based humanization selects human frameworks with the highest sequence similarity to preserve structural and functional integrity. Germline-based approaches use highly homologous human germline sequences, with selective back-mutations at key positions to balance immunogenicity and affinity. CDR-homology-based methods, by contrast, prioritize compatibility at the CDR level to enhance structural compatibility and reduce immune response. In all strategies, essential framework residues are usually retained to support antigen recognition [36, 74, 75].

Other techniques include resurfacing, introduced in 1992, which substitutes surface-exposed murine residues with human residues to reduce immunogenicity while preserving the native CDRs and framework [76]. SDR grafting represents another strategy, retaining only antigen-contacting residues in the CDRs and replacing the rest with human germline residues [77]. Although SDR grafting can yield highly human-like antibodies, it remains underexplored and has not yet produced approved therapeutics.

Among these methods, CDR grafting remains the most widely applied and clinically validated due to its balance of reduced immunogenicity and preserved specificity. Ongoing advances, including AI-assisted humanization, are expected to further improve the efficacy, safety, and structural compatibility of therapeutic antibodies (see Sect. "Antibodies from AI").

#### FDA-approved antibody drugs from hybridoma and humanization

Currently, 90 of the 144 FDA-approved mAb therapeutics on the market were generated using hybridoma technology (Table 1). Among these, 3 are murine mAbs, 15 are chAbs, and 72 are humanized antibodies. Notably, four of the top 10 best-selling antibody drugs in 2024 (Tables 2a, 2b) originated from hybridoma-based discovery platforms. These include pembrolizumab (1st best-selling antibody drug) for cancer immunotherapy, risankizumab (3rd) and ocrelizumab (8th) for autoimmune diseases, and vedolizumab (9th) for inflammatory bowel disease.

European rabbits (*Oryctolagus cuniculus*) have long been recognized as an important model organism in immunology and a valuable resource for generating commercial monoclonal and polyclonal antibodies. However, relatively few FDA-approved therapeutic antibodies have originated from rabbits. Instead, rabbit-derived antibodies are predominantly used in clinical diagnostics. For example, Roche's rabbit mAb anti-HER-2/neu (4B5) is utilized in immunohistochemical staining to confirm HER2-low status in breast cancer prior to treatment with trastuzumab deruxtecan [78]. In 2024, the FDA further approved this antibody as a companion diagnostic for the patients with HER-positive biliary tract carcinoma who might be eligible for zanidatamab treatment.

In 2019, the first humanized mAb originally derived from rabbit, brolucizumab (Beovu), was approved by the FDA. It is a scFv used to treat exudative (wet) age-related macular degeneration (AMD), diabetic macular edema, and macular edema caused by retinal vein occlusion by inhibiting vascular endothelial growth factor (VEGF) [79]. In 2020, the second rabbit-derived mAb drug, eptinezumab (Vyepti), was approved. Eptinezumab targets calcitonin gene-related peptide (CGRP) and blocks its interaction with receptors to prevent migraines [80].

### Antibodies from phage display

Phage-displayed antibody libraries have revolutionized antibody development, particularly in cancer treatment. Moreover, internationally renowned research institutions such as the MRC Laboratory of Molecular Biology in the UK [81], the German Cancer Research Center [82], and the Scripps Research Institute in the United States [83] have effectively implemented phage-displayed libraries, significantly contributing to advancements in antibody discovery. As key patents have expired, the incorporation of phage-displayed antibody technology could accelerate the development of human therapeutic antibodies for oncology and emerging infectious disease treatments [36].

Convenience of handling of a phage-displayed antibody platform, such as library construction, biopanning, screening, identification, and IgG production, are carried out in vitro and can be performed in a routine molecular biology laboratory (Fig. 3B) [84, 85]. Furthermore, these processes may be enhanced with automation systems for high-throughput manipulation. Based on this flexibility, we expect that phage display will become an indispensable means of antibody identification in the future, especially in combination with cutting-edge AI, big data analysis, deep learning, and antibody design [86, 87].

#### Human scFv and Fab libraries

Currently, two types of antibody fragments are regularly used for phage display, scFvs and Fabs. Both scFvs and Fabs contain six hypervariable CDRs, which provide a large contiguous surface for antigen recognition. However, a phage-displayed Fab typically exhibits a more stable structure than an scFv due to the additional CH1 and CL domain interface. Consequently, conversion of a Fab into an IgG configuration is less likely to lead to impaired binding affinity [88].

#### Naïve and synthetic antibody libraries

A naïve antibody library is constructed by adapting Ig gene sequences from the cDNA of B cells derived from peripheral blood of donors for use in phage-displayed hAb libraries. Initially, VH and VL genes are amplified by polymerase chain reaction (PCR) using primer sets derived from IgM genes. To construct an scFv-format antibody library, VL-linker-VH gene fragments are assembled through PCR-mediated procedures and subsequently cloned into phage display phagemid vectors [85]. Similarly, the Fab antibody library is built by assembling VL-CL and VH-CH1 gene fragments into phagemid vectors [89]. However, the construction of a naïve antibody library may be held back by ethical concerns, regulatory challenges, and limited availability of materials.

Construction of synthetic antibody libraries is based on knowledge-driven design of CDR sequences. Three key principles guide the design of synthetic antibody libraries: (1) Utilization of protein structure databases of antibody-antigen complexes to identify hotspot positions of contact residues; (2) Performance of sequence alignment using an Ig sequence database to define the displayed framework and CDR lengths in the library; (3) Calculation of amino acid frequencies at each hotspot to design CDR variation repertoires.

After library primer sets are synthesized using reverse genetics based on the designed CDR variation repertoires, the synthetic library can be constructed using the Kunkel protocol, yielding combinatorial complexity of more than 10^11^ variants [90].

#### Biopanning strategy to develop specific antibodies

Biopanning of phage-displayed antibody libraries can be performed with antigens coated on ELISA plates or magnetic beads, or with cells expressing the target antigen on the surface [91]. Of note, antigens immobilized on magnetic beads can also be adapted for automated and high-throughput panning using a Kingfisher instrument. The targeting antigens of biopanning may be purified proteins, carbohydrates, small molecule drugs, peptides, toxins, or protein conformational structures [19]. Notably, binders for bio-toxic drugs, toxins, or labile conformational structures cannot be obtained through traditional animal immunization techniques, but these antigens can be utilized with panning approaches. In one prominent example, panning was utilized to develop a COVID-19 rapid test. Strikingly, antibodies against the nucleoprotein and spike proteins of SARS-CoV-2 were panned and cloned for IgG production within one month [92, 93]. Phage display is one of technologies that was used to successfully generate antibodies in such a short timeframe.

#### Antibody optimization

Phage-display antibody libraries serve not only as a robust platform for antibody discovery, but the technology can also be leveraged as a powerful tool for antibody refinement. Optimization can be performed to enhance binding affinity, thermal stability or solubility, or it can be performed to reduce antigenicity [84, 94].

#### Nanobody library

In the 1990s, Raymond Hamers' team discovered a unique conformation of antibodies in camelids [95]. Unlike conventional antibodies, this type of antibody consists only of a heavy chain and lacks a light chain; thus, they are called heavy-chain-only antibodies (HCAbs) [96]. Similar HCAbs have also been found in cartilaginous fish, such as sharks [97]. The single antigen-binding domains of HCAbs in camelids are known as VHHs and in cartilaginous fish are called Variable New Antigen Receptor (VNARs). Both VHHs and VNARs are considered to be nanobodies (Nbs). Since the molecular weight of a Nb is only 15 kDa [98], these molecules generally exhibit better biodistribution and higher stability compared to conventional antibodies. Therefore, the potential applications of Nbs in therapy and diagnostics are highly promising.

Antigen-specific Nbs can be obtained from immune, naïve or synthetic libraries. Immune libraries may be derived from B cells of immunized camelids or sharks, and Nb gene libraries can subsequently be amplified using RT-PCR. In contrast naïve libraries are established using B cells from camelids or sharks without prior immunization, while synthetic libraries are created by designing random-mutant Nb sequences. These Nb libraries are transfected into *E. coli* and commonly screened using phage display or yeast display technologies to identify candidate Nbs for further development [98].

Besides their advantages in biodistribution and stability, the smaller size of Nbs improves the chance of binding sterically hindered epitopes, as compared to conventional antibodies. Therefore, Nbs carry high potential for development as therapeutic drugs. Caplacizumab (Cablivi) is the first FDA-approved VHH-based drug in 2019 for the treatment of aTTP. Caplacizumab was designed as a bivalent VHH molecule that specifically targets the A1 domain of vWF to prevent abnormal platelet aggregation. [99]. Ozoralizumab (Nanozora) was developed as a trivalent anti- TNFα Nb for the treatment of autoimmune diseases and was approved for treatment of rheumatoid arthritis in Japan in 2022 [100]. In the oncology domain, Nbs may serve as alternatives to conventional antibodies in different types of immunotherapies, such as multi-specific antibodies, ADCs, and CAR-T cell therapies [101, 102]. Nbs can also be used to treat infectious diseases. During the COVID-19 pandemic outbreak, multiple Nb-based therapies were developed [103]. These Nbs specifically bind to the viral Spike protein, disrupting its interaction with the ACE2 receptor and thereby exerting a therapeutic effect. In addition, Nb-based therapies targeting viral infections, such as influenza virus, RSV, norovirus and HIV, are under development [104–106]. Additionally, numerous candidate Nb drugs are being developed for the treatment of Parkinson’s disease, Alzheimer’s disease, and autoimmune disorders [102].

Compared to conventional antibodies, Nbs offer several practical advantages, including lower immunogenicity, enhanced thermostability, improved solubility, and superior distribution properties. However, a major limitation of Nbs is their short half-life in vivo, which affects therapeutic efficacy. Various modification strategies, such as Fc domain fusion or carrier protein binding, have been explored to extend the half-life of Nbs. Nevertheless, such modifications may alter the fundamental characteristics of Nbs. Despite these challenges, Nbs continue to demonstrate significant potential for future medical applications.

#### FDA-approved antibody drugs from phage display

To date, there have been 16 therapeutic antibodies developed with phage display approved by the FDA, each of which targets a key antigen associated with cancer or infectious disease (Table 1). Recently, marstacimab (Hympavzi), a first-in-class tissue factor pathway inhibitor (TFPI) antagonist for hemophilia A and B, was approved in 2024. In the same year, cosibelimab (Unloxcyt), a human IgG1 against PD-L1, was approved for cutaneous squamous-cell carcinoma. Most recently, garadacimab (Andembry) is the first FDA-approved factor XIIa inhibitor, received approval in 2025 for hereditary angioedema, providing a once-monthly prophylactic therapy.

In summary, antibody development with phage-displayed library technology integrates automated screening processes with high-throughput functional analysis. Consequently, deep learning and AI can be utilized in antibody design, as systematic validation can be performed with automated high-throughput phage display technology. This makes the platform a promising ground for advancing AI-driven antibody development [52].

### Antibodies from human Ig transgenic animals

Transgenic animal models offer an in vivo approach to generating fully human mAbs similar to in vitro phage-displayed libraries, but the animals have higher antibody repertoire than phage-displayed libraries (Fig. 3C). Nevertheless, developing transgenic mice is challenging due to the difficulty of inserting large human Ig genes into mouse genomes. These genes must express human variable (V), diversity (D), and joining (J) segments in mouse, which closely mimic the human immune system.

#### Fully human antibody mice

In 1985, the concept of using transgenic mice to produce hAbs was first proposed. Alt et al. suggested that the insertion of human Ig genes into the mouse germline could allow researchers to produce new mAbs with human sequences [107]. In 1989, Brüggemann et al. introduced an unrearranged human heavy-chain minilocus, consisting of V, D and J segments along with the mu-chain (Cμ) constant region into the germline of mice. As a result, the human IgM gene was able to be rearranged and expressed in the B lymphocytes of the transgenic mice [108]. In 1992, Taylor et al. generated a new transgenic mouse line carrying an unrearranged germline human heavy chain and light chain minilocus. The human heavy chain transgene contained one functional VH segment, ten D segments, six JH segments and the Cμ and Cγ1 constant regions. Meanwhile, the human light chain transgene contained one functional Vĸ segment, five Jĸ segments and a Cĸ segment. Both human IgM and IgG1 were observed in serum samples from these transgenic mice, and the study revealed that antibody class switching from IgM to IgG1 could occur in this transgenic model [109]. Since less than 10% of all antibodies produced in the transgenic mice were hAbs, numerous endogenous Ig knockout mouse strains were created to eliminate interference from mouse Ig [109].

In 1993, Chen et al. knocked out murine JH and Jκ genes via gene targeted deletion, inactivating mouse Ig [110]. A human IgH and IgL transgenic mouse was then bred with a murine IgH and IgL knockout mouse to establish lines capable of generating a broader diversity of hAbs. In 1994, HuMab Mouse, the first hAb mouse, was developed via deletion of murine heavy and light chain genes and expression of human heavy chain and light chains [5]. This enabled the generation of fully human mAbs. However, only 80 kb of the human IgH genome was introduced into the mice [5]; the entire human IgH genome is approximately 1.25 Mb, and the Igκ genome is approximately 1.82 Mb.

Since the diversity of generated hAbs depends on the successful introduction of the human Ig genome into mice, yeast artificial chromosome (YAC) vectors were utilized to introduce large segments of the human IgH and Igκ genomes into mouse embryonic stem (ES) cells through yeast spheroplast-ES cell fusion [4, 111, 112]. In 1994, Green et al. used YAC vectors to introduce the human IgH (~ 220 kb) and Igκ (~ 170 kb) loci into the mouse germline, enabling the production of hAbs upon immunization. These transgenic mice were then bred with mice lacking endogenous heavy and κ chain genes to generate the XenoMouse [4]. In 1997, Mendez et al. further developed the XenoMouse II strain by introducing a large human IgH (~ 1,020 Kb) or Igκ (~ 800 Kb) YAC, including 66 VH and 32 Vκ genes, into ES cells of endogenous Ig-knockout mice, thereby enabling the production of fully hAbs [21].

Building on these advancements, in 2000, Tomizuka et al. developed TransChromo (TC) technology. This involved by introducing two transmissible human chromosome fragments (hCFs), one carrying the human IgH locus (~ 1.5 Mb, from chromosome 14) and the other carrying the human Igκ locus (~ 2 Mb, from chromosome 2), into an endogenous Ig-knockout mouse strain to create the TC Mouse [113]. However, the TC Mouse exhibited low hybridoma production efficiency**,** less than one-tenth of that observed in normal mice. To address this limitation, Ishida et al. (2002) subsequently developed the KM Mouse by cross-breeding the Kirin TC Mouse with a Medarex YAC-transgenic mouse [24]. While this resulting mouse line demonstrated reduced interference from endogenous mouse Ig and comprehensive coverage of human Ig genes**,** challenges persisted. Specifically, hAb production, Ig class switching, and somatic hypermutation remained inefficient due to the absence of mouse constant region gene expression [114].

#### Chimeric human antibody mice

To enhance mouse Fc-mediated signaling for somatic hypermutation during antibody affinity maturation and effector functions, it is essential to retain the endogenous mouse constant region gene. Thus, chimeric hAb mice were created with a human Fab region combined with a mouse Fc region [114–116]. In 2013, Osborn et al. developed a humanized rat strain, OmniRat, which carries a novel chimeric human/rat IgH locus consisting of human VH, D and JH segments linked to the rat CH locus, along with fully human IgL loci [25]. Following antigen immunization, the levels of antibody production, high-affinity serum IgG, somatic mutations, and B cell recovery were comparable to those of normal rats, and human V(D)J transcripts exhibited high diversity [25]. In 2014, Lee et al. developed KyMouse, a transgenic mouse carrying a human VH-D-JH repertoire linked to the mouse constant region. Following antigen immunization, KyMouse produced high-affinity antibodies with long, human-like CDR-H3, broad epitope coverage, and extensive somatic hypermutation [117].

Building on these advances, Murphy et al. employed bacterial artificial chromosome (BAC)-mediated targeting to sequentially replace 2.6 Mb of mouse IgH variable region and 3 Mb of mouse kappa light chain variable region with human counterparts, while maintaining the endogenous mouse constant regions [23]. This approach enabled the creation of the VelocImmune mouse platform. The retention of murine Fc regions ensures compatibility with murine immune effector mechanisms and allows for efficient in vivo selection and maturation of functional human-mouse chAbs.

#### FDA-approved antibody drugs derived from human antibody mice

Panitumumab, approved in 2006, was the first FDA-approved fully hAb derived from a transgenic mouse (XenoMouse). Since then, multiple transgenic animal platforms (mouse/rat) have been used to generate hAbs, including XenoMouse (Abgenix; acquired by Amgen in 2005), HuMAb Mouse (Medarex; acquired by Bristol Myers Squibb in 2009), KM Mouse (Kirin), VelocImmune (Regeneron), OmniRat (Open Monoclonal Technology; acquired by Ligand in 2016), KyMouse (Kymab; acquired by Sanofi in 2021), H2L2 Mouse (Harbour Antibodies), Trianni Mouse (Trianni; acquired by AbCellera in 2020), NeoMab (GemPharmatech), and RenMab (Biocytogen) [118] (Table 3). As of August 2025, thirty FDA-approved hAbs have been derived from these transgenic platforms (see Table 1).

**Table 3:** Human antibody transgenic animals in the world

Platform	Company	hVH^a^	hVK^b^	Constant	Country	mAb drugs approved by FDA	Reference	Structure
HuMab Mouse	Genpharm/Medarex/Bristol Myers Squibb	4	4	Human (Cμ)	U.S	13	1994 Lonberg et al	
XenoMouse	Abgenix/Amgen	17	17	Human (Cμ-Cδ-Cγ2)	U.S	11	1994 Green et al	
KM Mouse	Kyowa Kirin & Medarex	N.D	N.D	Human	JP & U.S	1	2002 Ishida et al	
VelocImmune	Regeneron	80	40	Mouse	U.S	8	2014 Murphy et al	
OmniRat	Open Monoclonal Technology/Ligand	22	12	Rat	U.S	0	2013 Osborn et al	
KyMouse	Kymab/Sanofi	43	37	Mouse	UK	0	2014 Lee et al	
H2L2 Mouse	Harbour Antibodies BV	18	11	Mouse	CN	0	https://harbourantibodies.com	
Trianni Mouse	Trianni/AbCellera	44	39	Mouse	U.S	0	https://trianni.com/	
RenMab	Biocytogen	44	38	Mouse	CN	0	2020 Ng et al	
NeoMab	GemPharmatech	N.D	N.D	Mouse	CN	0	https://en.gempharmatech.com/	

Human sequence,  Mouse sequence

^a^The number of human VH gene segments

^b^The number of human VK gene segments

N.D., not disclosed

Eleven FDA-approved antibodies were developed using XenoMouse, including Cosentyx, Vectibix, Prolia, Repatha, Lumicef, Imfinzi, Aimovig, Padcev, Tezspire, Imjudo and IMDELLTRA. Panitumumab (Vectibix,) targets EGFR as treatment for patients with metastatic colorectal carcinoma [119]. Denosumab (Prolia) binds to RANKL and prevents the activation of its receptor, RANK, on the surface of osteoclasts, thereby inhibiting osteoclast function and bone resorption. In 2010, this antibody was approved by the FDA for the treatment of osteoporosis [120]. In 2015, secukinumab (Cosentyx) was approved by the FDA for the treatment of moderate to severe plaque psoriasis. The mechanism involves selective binding to IL-17A, which blocks IL-17A–IL-17R interaction [121]. Durvalumab (Imfinzi) blocks PD-L1 binding to PD-1 and was approved in 2017 for the treatment of patients with locally-advanced or metastatic urothelial bladder cancer [122].

Thirteen antibodies derived from HuMAb Mouse have received approval from the FDA, including Darzalex, Stelara, Opdivo, Ilaris, Simponi, Arzerra, Yervoy, Zinplava, Tepezza, RYBREVANT, Saphnelo, TIVDAK, and Opdualag. In 2014, the FDA approved nivolumab (Opdivo) for the treatment of unresectable or metastatic melanoma. Nivolumab targets PD-1, blocking its interaction with PD-L1 and PD-L2 ligands. By inhibiting this pathway, nivolumab enhances T-cell activation, enabling the immune system to recognize and destroy cancer cells [123]. Ustekinumab (Stelara) targets the p40 subunit of the IL-12 and IL-23 cytokines to effectively reduce inflammation. It was approved in 2009 for the treatment of moderate to severe plaque psoriasis [124]. Daratumumab (Darzalex) is the first FDA-approved therapy targeting CD38 protein. This drug is administered subcutaneously for the treatment of patients with multiple myeloma [125].

Eight antibodies developed using VelocImmune mice have been approved by the FDA, including Dupixent, Praluent, Kevzara, Libtayo, Inmazeb, Evkeeza, VEOPOZ and Lynozyfic. A prominent example is dupilumab (Dupixent), which binds to IL-4 receptor alpha, inhibiting the IL-4 and IL-13 pathways. It was approved in 2017 for the treatment of moderate to severe atopic dermatitis [126]. Burosumab (Crysvita) is the only FDA-approved antibody derived from KM mouse. It was approved in 2018 for the treatment of X-linked hypophosphatemia and acts by blocking the function of fibroblast growth factor 23 (FGF23), increasing renal tubular phosphate reabsorption and normalizing serum phosphate levels [127]. Importantly, transgenic mice can be integrated with mouse hybridoma or single B cell technologies to further enable the generation of high-affinity hAbs.

### Antibodies from single B cell antibody technology

Single B cell antibody technology is an advanced means of developing fully hAbs in which antigen-specific B cells are isolated. This approach offers several advantages, including generating fully hAbs, naturally paired heavy and light chains, a rapid discovery process, and the ability to identify rare antibodies (Fig. 3D) [36, 128]. Using single B cell screening, it may only take three to four weeks to identify lead sequences for further development. These substantial benefits of accelerated therapeutic antibody discovery and drug development are especially relevant for infectious disease and were particularly beneficial during the COVID-19 pandemic [129]. Most SARS-CoV-2 neutralizing antibodies granted Emergency Use Authorization (EUA) in the U.S. were generated using this critical technology [27, 35]. The first marketed SARS-CoV-2 neutralizing antibody, bamlanivimab, was identified in February 2020 and received EUA from the FDA in November 2020, after less than one year of development [34].

The technology involves direct amplification of the genes encoding VH and VL regions from single B cells, maintaining native pairing original binding properties. The process includes B cells isolation, VH/VL identification cloning, expression, screening and characterization.

*(1) B cell isolation*. Splenocytes or peripheral blood mononuclear cells (PBMC) from infected or immunized subjects with a specific antigen are isolated. Antigen-specific memory B cells can be sorted into individual wells via fluorescence-activated cell sorting (FACS). The method is simple, fast and high-throughput, and various isolation protocols have been developed for different species [130–135].

For human B cells, commonly used immortalization methods include Epstein-Barr virus (EBV) transformation, Simian virus 40 (SV40) virus infection, in vitro genetic modification, and activating CD40 [136]. The FDA-approved anti-Ebola antibody, ansuvimab, was successfully developed from a survivor’s memory B cells and identified using an EBV-based immortalization strategy [26]. However, single-cell culture or B cell immortalization is costly and time-consuming, direct VH and VL genes identification from single B cells has emerged as a more efficient strategy for mAb screening.

*(2) Identification of VH and VL genes*. VH/VL sequences from single B cell RNA are obtained by RT-PCR using specific primers, followed by nested or semi-nested multiplex PCR. Primer design is critical, as it affects the recovery efficiency of immunoglobulin variable regions. As such, primer sets have been developed for different species [7, 8, 130, 135].

Recently, single-cell transcriptomic strategies employing barcode-labeled beads and NGS have enabled large-scale identification of antigen-specific antibody sequences. As these approaches generate vast repertoires, it is impractical to express and experimentally validate every sequence. Therefore, several criteria have been proposed to prioritize candidates with higher likelihood of functional activity; these include clonal frequency, the extent of somatic-hypermutations (SHMs), the length of CDR-H3, and comparison to previously identified antigen-specific antibody clones [137–140].

#### FDA-approved human antibodies derived from human B cells

Single B cell technology has demonstrated great potential for rapidly screening specific antibodies against novel emergent pathogens. This approach enables isolating potent antibodies not only from samples subjected to long-term cryopreservation but also from convalescent patients many years after infection. Thus, this method has extensive applications in antibody drug development for infectious disease.

For example, the cross-neutralizing SARS-CoV-2 antibody sotrovimab (S309) was identified from a blood sample collected from a SARS-CoV-infected individual in 2013 [141]. Another notable example is ansuvimab (mAb114), a fully hAb against Ebola virus, derived from the memory B cells of two survivors collected more than 11 years after their initial infection [26]. Importantly, ansuvimab exhibits superior efficacy in reducing mortality from Ebola virus disease compared to remdesivir and ZMapp (a triple mAb agent) [142]. It was approved by the FDA in December 2020 for treating Ebola virus infection in adults and children.

Another notable B cell derived mAb is nirsevimab (MEDI8897), which neutralizes RSV by targeting the prefusion structure of RSV F protein. Nirsevimab was engineered from D25, a human IgG1 mAb originally isolated from memory B cells of human donors [143]. It incorporates YTE substitutions in the Fc region to extend its half-life [144], allowing a single intramuscular injection to provide protection during the RSV season. A single dose effectively protects healthy infants from RSV infection, associated hospitalizations and severe disease [145]. In July 2023, the FDA approved nirsevimab for the prevention of RSV in infants and some young children.

Subsequently, another RSV neutralizing antibody, clesrovimab (MK-1654), was isolated from the memory B cells of RSV-infected individuals [146]. According to Merck’s announcement in October 2024, results from a phase IIb/III trial (NCT04767373) indicated that clesrovimab significantly reduced RSV-associated hospitalizations and RSV-associated lower respiratory tract infection hospitalizations by over 84% and 90%, respectively, over a five-month period. Based on these compelling data, the FDA granted approval to clesrovimab in June 2025.

Antibodies isolated from B cells are not only applicable to infectious diseases but also to other fields. For example, aducanumab (BIIB037) is a hAb targeting beta amyloid, which was derived from a blood lymphocyte library collected from elderly individuals [147]. Aducanumab was approved by the FDA in 2021 for the treatment of Alzheimer’s disease, making it the first fully hAb approved for this indication.

#### Clinical development of single B cell-derived antibodies

Due to the key advantage of a rapid discovery process, major clinical development efforts are underway to evaluate single B cell-derived antibodies for infectious diseases. Aside from direct isolation from infected individuals, another efficient approach to identify antibodies from B cells is to screen for potential therapeutic antibodies after vaccination [148–150]. For example, the anti-malaria antibodies CIS43LS and L9LS are modified from the original CIS43 and L9 antibodies, which were derived from B cells of vaccinated volunteers [148, 151]. CIS43LS has been evaluated in a completed phase II clinical trial (NCT04329104), which showed that a single intravenous infusion of 10 mg/kg exhibited 75% protective efficacy against *P. falciparum* infection over a 6-month malaria season [152]. Similarly, L9LS has been evaluated in three phase II trials (NCT05304611/NCT05400655/NCT05816330) for children and adults [153]. Recently, potent antibodies capable of preventing infection by antibiotic-resistant *Staphylococcus aureus* have also been identified from the B cells of a participant in a phase I vaccine trial [150]. These studies demonstrate the versatility and speed of antibody screening and therapeutic discovery processes using human single B cells.

Acquired human immunodeficiency syndrome (AIDS) remains a major public health issue, as approximately 39.9 million people worldwide are living with HIV. Advances in single B cell technologies have facilitated the discovery of numerous broadly neutralizing antibodies [154], several of which are under evaluation in clinical trials on monotherapies and combination therapies [155, 156]. For instance, VRC01, 3BNC117 and N6LS monotherapies have been assessed in phase II trials (VRC01, NCT02716675/NCT02568215; 3BNC117, NCT02446847; N6LS, NCT04871113). Notably, it was also shown that N6LS administered every four months in combination with monthly cabotegravir long-acting (CAB LA) can effectively maintain undetectable viral load [157].

Many serious bacterial infections are caused by biofilm formation, which plays a major role in recurrent infections and antibiotic resistance. TRL1068 was identified through single B cell screening and targets a bacterial biofilm anchoring protein. Strikingly, this antibody has been shown to restore bacterial sensitivity to antibiotics [158]. TRL1068 may therefore serve as an effective adjuvant to antibiotic therapy, particularly for the treatment of infections caused by biofilm-producing antibiotic-resistant bacteria [159]. Recently, a phase II clinical trial has begun recruitment of patients with prosthetic joint infection for TRL1068 treatment (NCT06621251).

Following the approval of anti-Ebola and anti-RSV antibodies by the FDA, single B cell approaches have gained increasing attention for antibody drug development. In particular, human single B cell technology has been utilized with remarkable speed and efficiency in the discovery of therapeutic antibodies against COVID-19. However, due to the high mutation rates of RNA viruses (e.g., SARS-CoV-2 and influenza virus), the identified neutralizing antibodies often have significant limitations due to immune escape, which poses a major obstacle to clinical development. Nevertheless, the integration of this technology with hAb transgenic animal platforms and NGS is expected to broaden the application of single B cell screening for development of novel diagnostics and targeted antibody therapies across various domains. In one recent example of this trend, T-cell receptor (TCR) mimetic antibodies were developed by screening single B cells from immunized VelocImmune mice for use in a CAR-T cell therapy [160].

### Antibodies from AI

The need for extensive experimental screening, high costs, and long development timelines often limit traditional antibody discovery. Identifying functional antibodies with high specificity and affinity requires navigating a vast combinatorial search space, which is both time-consuming and resource-intensive. The availability of large-scale protein structure and sequence datasets has played a key role in enabling the training of modern AI models. These datasets have provided the foundation to model antibody structure, sequence diversity, and antibody-antigen interactions. Recent progress in this area has significantly accelerated antibody design, reducing the timeframe from initial design to experimental validation to weeks or months. This acceleration has been associated with a more than tenfold decrease in costs. AI-driven antibody design technologies may be categorized into five key areas: antibody structure prediction, sequence generation, de novo antibody design, antibody-antigen complex prediction, and antibody developability assessment (Fig. 3E).

Most of AI-related advances in antibody development have been driven by the availability of large-scale antibody sequence and structure datasets, such as those provided by the Observed Antibody Space (OAS), SAbDab, and the Protein Data Bank (PDB) [161–163]. However, these resources are primarily populated by human-derived repertoires, common pathogens (e.g., SARS-CoV-2), and Fv-region structures. As a result, AI models trained on these datasets may exhibit biases and limited generalizability when applied to non-hAb frameworks, rare epitopes or full-length antibodies. Addressing these limitations will be critical for expanding the utility of AI-driven design across diverse immunological settings.

#### Antibody structure prediction

Predicting the three-dimensional structure of antibodies, particularly for the Fv, is crucial for understanding paratope-epitope interactions and guiding rational antibody design. Experimental structure determination techniques such as X-ray crystallography and cryo-electron microscopy, are accurate but labor-intensive and low-throughput. To overcome these limitations, computation-based methods provide alternatives for downstream engineering and development strategies. Among the six CDRs, the third heavy-chain loop (CDR-H3) remains the most difficult to predict accurately. Unlike other CDRs that often adopt canonical structures, H3 exhibits high structural variability and lacks consistent motifs, making it a key challenge in computational antibody modeling. This challenge may be overcome with IgFold [164], which combines pre-trained antibody-specific language models with a graph Transformer to directly predict entire variable region structures from sequences.

In a comparative evaluation of four deep learning methods (i.e., IgFold, DeepAb, ABlooper, and AlphaFold-Multimer [165–167]), IgFold achieved the highest average accuracy in predicting the CDR H3 loop, highlighting its strength for modeling this structurally diverse and challenging region. Another important tool is ImmuneBuilder [168], an improved framework based on AlphaFold2-multimer that is optimized for antibody, nanobody and TCR modeling. It uses an ensemble of four AlphaFold2-multimer-based models and refinement steps to predict Fv structures and provides per-residue error estimates by predicting structure ensembles. In a benchmark evaluation of recently solved antibody structures, ImmuneBuilder achieved an average RMSD of 2.81 Å on the CDR H3 region, slightly outperforming AlphaFold-Multimer (2.90 Å), while providing significantly faster predictions. While IgFold and ImmuneBuilder can both be used to accurately predict antibody Fv structures, their design strategies differ. IgFold prioritizes speed and efficiency by removing the need for multiple sequence alignments, making it suitable for large-scale predictions. In contrast, ImmuneBuilder emphasizes accuracy and estimates per-residue error through model ensembles. IgFold prioritizes scalability and efficiency, whereas ImmuneBuilder provides enhanced accuracy and residue-level error estimates. Both approaches highlight the growing utility of AI-driven antibody modeling.

#### Antibody sequence generation

Large language models (LLMs) are deep learning architectures trained to capture statistical patterns, contextual relationships, and functional motifs from large-scale sequence data. Several LLMs have been developed to model protein or antibody-specific sequences; these include ESM, ProBERT and AntiBERTa [169–171]. These models can be utilized for various applications, including protein structure prediction, variant effect estimation, and antibody repertoire modeling. When fine-tuned or explicitly designed for antibodies, these tools can model structural features and evolutionary constraints with high specificity. When trained on antibody or immune repertoire data, the models enable in silico generation of antibody sequences with desired features such as framework compatibility, CDR diversity and developability. The LLMs further support tasks such as full-sequence generation, conditional infilling of hypervariable regions, or sampling diverse candidates for downstream screening. In contrast, traditional approaches often rely on labor-intensive screening or random mutagenesis, which are limited in scale and efficiency.

LLM-based generative models now provide data-driven alternatives for exploring antibody sequence space and enabling targeted design through learned sequence priors. IgLM introduces a Transformer-based language model specifically designed for antibody sequence infilling, enabling targeted design within existing frameworks [172]. Its application is beneficial when applied to affinity maturation workflows, wherein specific CDR loops (especially H3) are diversified while preserving overall framework compatibility. In terms of practical benchmarks, CDR-H3 sequences generated by IgLM showed improved in silico developability profiles, including lower aggregation propensity and higher humanness scores. IgLM excels at producing variants suitable for affinity maturation processes but requires subsequent filtering or experimental validation to ensure binding functionality. AntiBERTa is a BERT-based language model trained on large-scale natural antibody repertoires to generate or assess sequences by iterative residue prediction or controlled sampling [173]. It has been applied to evaluate the humanness and functional plausibility of synthetic antibodies, and it supports controlled sampling strategies that preserve natural-like sequence motifs. Together, these models can effectively predict suitable antibody-specific motifs, providing diverse and optimized candidates for wet-lab screening or further in-silico filtering.

#### Antibody-antigen complex structure prediction

While antibody structure prediction focuses on modeling the Fv region of the antibody alone, predicting the structure of antibody-antigen complexes involves modeling the interaction interface. This information is critical for predicting epitope recognition and guiding rational antibody design. Traditional approaches such as molecular docking and template-based modeling have provided valuable insights but are limited by template availability and computational complexity. Recent advances in deep learning have enabled more accurate and scalable antibody-antigen interaction predictions, even without high-quality structural templates. AlphaFold-Multimer, an extension of AlphaFold2, has demonstrated strong performance in predicting protein-protein complex structures, including antibody-antigen pairs. While AlphaFold-Multimer was not explicitly trained on antibody-antigen complexes, it can still produce accurate models when paired with proper input formatting and careful sequence curation. However, its predictions may be biased by evolutionary features such as conserved regions in antigens or dominant binding modes in training data.

A prominent example of this approach is provided by Gao and Skolnick, who used AF2Complex, a deep learning tool, to predict antibody-antigen interactions focusing on the SARS-CoV-2 spike RBD [174]. Three different MSA strategies were tested, and the combined strategy showed the best performance. When used for prediction of 36 known antibody-RBD structures, the method achieved a 61% recall and a 47% success rate. In a more extensive set of ~ 1,000 antibodies, the RBD-binding strategy identified 25% of true binders with 90% precision, including as few as 60 RBD-targeting sequences in MSAs improved prediction accuracy, suggesting practical potential for antibody discovery. AlphaFold 3 (AF3) shows substantially higher accuracy in antibody-antigen interface prediction than AlphaFold-Multimer v2.3 [50]. It uses a diffusion-based generative model to directly predict atomic coordinates, allowing more flexible modeling of complex interfaces. The model benefits from extensive sampling, with prediction quality increasing to 1,000 seeds per target. AF3 achieves higher DockQ scores on low-homology antibody-antigen benchmarks, indicating better interface prediction. These advances demonstrate the potential of AF3 for accurate modeling of immune interactions without heavy reliance on co-evolutionary signals. Overall, AI-based antibody-antigen complex prediction models can enable the transition from template-dependent docking to accurate, data-driven modeling. Training datasets are growing in diversity and quality, while attention mechanisms and geometric learning continue to improve. Thus, these models are expected to play an increasingly central role in epitope-specific antibody design and affinity maturation strategies.

#### De novo antibody design

De novo antibody design involves the computational generation of antibody sequences and structures from scratch, without relying on existing antibody templates or prior experimental structures. Unlike traditional antibody discovery (screening natural repertoires), synthetic libraries and immunized libraries, de novo design leverages generative models to directly create novel antibodies with specific structural or functional properties. This approach is promising for targeting challenging antigens or epitopes lacking known binders. DiffAb is an antigen-specific joint antibody structure and sequence design diffusion model [175]. It generates new CDR sequences and structures conditioned on a predefined antibody-antigen complex, making it suitable for tasks where a target interaction geometry is already known. In its design workflow, the antibody-antigen complex must be provided as an initial input. The original CDRs are removed from the input and replaced by randomized starting points. Then, the model iteratively refines the CDR regions to produce novel variants that are predicted to interact with the given antigen. While powerful, this approach is limited by the prerequisite of an accurate complex structure for use as an input. This limitation may restrict use of the tool in early discovery stages, when structural information is generally lacking. RFdiffusion is a diffusion-based generative framework for protein design, notable for designing novel binders [52]. RFantibody is a refined version of RFdiffusion, which has been successfully utilized to produce a binder for a predefined epitope of the antigen *Clostridium difficile* toxin B (TcdB) [176]. Despite its generative capability, RFantibody still requires a high-resolution epitope structure input as well as careful control over scaffold positioning to ensure functional binding.

#### Antibody developability assessments

##### Antibody humanization

Recent advances in AI have yielded data-driven strategies that streamline and enhance the humanization process. For instance, Hu-mAb applies a supervised machine-learning approach for sequence humanization based on large repertoire data [177]. The model recommends mutations that increase humanness while maintaining critical structural features. BioPhi is another integrated platform for humanness evaluation and antibody humanization using deep learning [178]. The model provides humanness scoring and mutation guidance, enabling the automated refinement of non-hAbs into human-compatible variants. Among recent developments, AbNatiV offers fine-grained residue-level assessments of antibody nativeness by comparing sequences to high-quality hAb datasets [179]. It generates interpretable metrics of humanness and immunogenicity risk, allowing users to identify and selectively modify non-human-like residues with minimal impact on antibody function.

AI-guided humanization strategies represent a substantial improvement over traditional heuristic-based methods. By enabling targeted, interpretable and automatable design, these models contribute to faster and more reliable development of therapeutic antibodies with reduced immunogenic profiles. As these tools continue to integrate structural modeling, immunogenicity prediction and experimental feedback, they are expected to deliver robust, automated pipelines for therapeutic antibody humanization.

##### Other developability assessments

In addition to Ab humanization, other important aspects of developability include immunogenicity, thermostability, solubility and aggregation propensity, all of which impact manufacturability and in vivo performance. Immunogenicity refers to the potential of an antibody to elicit an unwanted immune response. This attribute is a critical consideration in therapeutic development. While humanization reduces immunogenicity by increasing sequence similarity to native hAbs, more direct immunogenicity prediction tools are being developed. AbImmPred is a deep learning-based model that predicts immunogenicity based on the variable regions of antibodies [180]. It uses pre-trained antibody language models and automated machine learning frameworks to enable early-stage filtering of candidate sequences that may carry higher immunogenic risk, complementing existing humanization pipelines. Solubility and aggregation propensity are key developability attributes influencing antibody expression and formulation. CamSol is an AI-based computational model that assess antibody solubility and aggregation propensity from sequence alone [181]. CamSol accurately predicts aggregation hotspots, significantly assisting in identifying and mitigating developability risks early in design processes. Finally, therapeutic Antibody Profiler (TAP) evaluates an antibody on a range of (sequence-based) liabilities, including CDR lengths, patches of surface hydrophobicity, and charge [182]. TAP offers a holistic scoring approach that integrates multiple biophysical metrics, supporting early-stage triage of antibody candidates.

#### Integrated and multi-objective antibody design

Although AI-based tools have significantly enhanced individual stages of the antibody design pipeline, uncertainty may accumulate by the sequential application of multiple predictive tools, potentially reducing the overall success rate. This limitation necessitates the development of integrated design approaches that simultaneously consider multiple developability and functional criteria during antibody generation. Recent studies have proposed frameworks that explicitly address multi-objective optimization in antibody design. AB-Gen is a generative framework combining a pre-trained language model with reinforcement learning to design antibody libraries that simultaneously optimize for multiple developability attributes, including high binding affinity, reduced immunogenicity, and improved physicochemical properties. It can generate diverse and therapeutically viable antibody sequences by integrating these objectives into the generation process[183]. AntBO applies combinatorial Bayesian optimization to design CDR-H3 loops that balance binding affinity with favorable biophysical properties, achieving strong performance with a small number of experimental evaluations [184]. These methods are representative of an important trend toward holistic antibody design in which developability, specificity, stability and manufacturability are optimized concurrently rather than in disconnected stages. As the accuracy and coverage of generative and predictive models improve, these integrative approaches can potentially increase design success rates and accelerate therapeutic antibody development.

AI has emerged as a transformative force in antibody discovery and engineering, with different models now supporting tasks across the design spectrum, including structure prediction, sequence generation, de novo binder creation, complex modeling, and developability assessment. Tools such as IgFold and ImmuneBuilder facilitate rapid structural modeling, while LLMs like IgLM and AntiBERTa offer data-driven frameworks for antibody sequence design. Diffusion-based models like DiffAb and RFantibody enable epitope-guided binder generation, and tools like TAP, CamSol and AbImmPred provide essential developability filters. Despite these advances, most methods are optimized for isolated tasks and often operate under idealized assumptions. The integration of these models into full antibody discovery pipelines remains hindered by the compounding of predictive uncertainty and limited availability of experimental verification. Only a few models (e.g., RFantibody and DiffAb) have clear wet-lab validation, highlighting a crucial gap between in silico design and real-world success. Emerging multi-objective frameworks, including AB-Gen and AntBO, aim to address these challenges by concurrently optimizing for binding efficacy, stability, immunogenicity and manufacturability. These tools represent important progress toward end-to-end generative platforms for therapeutic antibody design. While AI-powered antibody design has achieved impressive coverage and flexibility, its full potential will only be realized through tighter coupling with experimental workflows, robust benchmarking, and the development of integrative, task-aware systems with demonstrated capability to generate safe and effective therapeutic antibodies.

### Therapeutic antibodies with engineered Fc region

The Fc region of IgG contains the CH2 and CH3 domains of the heavy chain, which are constant regions. Modifications to the Fc region are widely applied in therapeutic antibodies and bispecific IgG-based heterodimeric antibodies to enhance their pharmacokinetic, pharmacodynamic and effector characteristics. Up to now, Fc engineering strategies in FDA-approved therapeutic antibodies have been utilized for half-life extension and modulation of antibody-dependent cellular cytotoxicity (ADCC) and complement-dependent cytotoxicity (CDC).

#### Engineering the Fc region to extend half-life

The neonatal Fc receptor (FcRn) mediates transcytosis of maternal IgGs across mucosal barriers to offspring and plays a critical role in maintaining high IgG concentrations in circulation [185]. FcRn is involved in the intracellular vesicular trafficking network, particularly within acidic endosomes, where it facilitates the recycling of IgG and albumin [186]. The receptor binds to IgG under acidic conditions (pH 6.0-6.5) and dissociates at neutral to mildly alkaline pH (pH 7.0-7.5) [187]. Several therapeutic antibodies have engineered Fc regions that increase FcRn affinity, thereby reducing lysosomal degradation and enhancing recycling.

##### YTE variant

The M252Y/S254T/T256E (YTE) substitution has been applied in the development of several therapeutic antibodies by AstraZeneca to extend serum half-life. This modification can be used alone or in combination with other Fc variants, including YTE-FES and YTE-KF (described below). The two prophylactic mAbs, nirsevimab (Beyfortus) and clesrovimab (Enflonsia), both carrying the YTE modification, provide long-acting protection against RSV-associated lower respiratory tract infections, with half-lives of 89 and 80 days, respectively. They were approved by the FDA in 2023 and 2025, respectively [188]. In addition, the YTE modification results in an 8.3-fold reduction in binding affinity to FcγRI and a 1.8- to 2.5-fold reduction in binding to FcγRIIA and FcγRIIIA, compared with a non-YTE-modified control. These findings suggest that YTE modification leads to a modest reduction in Fcγ receptor binding affinity [189].

##### YTE-FES variant

The YTE modification has also been integrated into prophylactic SARS-CoV-2 mAbs, i.e., tixagevimab and cilgavimab (Evusheld), which received EUAs from the FDA from December 2021 to January 2023. These antibodies feature dual Fc modifications: the YTE modification increases FcRn affinity and extends serum half-life, while L234F/L235E/P331S (collectively referred to as FES or TM mutations) reduces binding to Fcγ receptors and C1q in order to mitigate risk of antibody-dependent enhancement (ADE) *(described in the LALA variant section)*. The mean serum half-lives are 87.9 days for tixagevimab and 82.9 days for cilgavimab [190]. However, concerns remain regarding YTE-FES constructs, including reduced thermostability and a higher propensity for aggregation [191–193]. Beyond prophylactic antiviral applications, the FES modification has also been employed independently to dampen Fc-mediated effector functions in therapeutic antibodies for autoimmune diseases.

##### LS variant

The LS variant (Xencor’s Xtend™) consists of M428L/N434S mutations in the Fc region. This variant was developed to enhance IgG binding to FcRn at acidic pH, thereby extending serum half-life without increasing binding at neutral pH. The LS variant has been incorporated into several therapeutic mAbs, including ravulizumab, which was approved by the FDA in 2018 for paroxysmal nocturnal hemoglobinuria, demonstrating a serum half-life of ~ 49.7 days. Sotrovimab, an anti-SARS-CoV-2 antibody with the LS variant, received an EUA for COVID-19 treatment from May 2021 to April 2022. In cynomolgus monkeys, the LS modification extended the half-life of cetuximab from 2.9 to 13.9 days (~ 4.8-fold) and bevacizumab from 9.7 to 31.1 days (~ 3.2-fold) compared to unmodified counterparts [194]. The LS modification has been shown to facilitate FcγR and C1q interactions, thereby preserving effector functions such as ADCC and CDC.

#### Engineering the Fc region to block FcRn-mediated IgG recycling

*YTE-KF variant:* Efgartigimod (Vyvgart) is a first-in-class FcRn antagonist comprising a human IgG1-derived CH2-CH3 Fc fragment (residues 220-447). It features a YTE-KF variant (M252Y/S254T/T256E/H433K/N434F), which enhances FcRn binding at both acidic and neutral pH, thereby disrupting FcRn-mediated IgG recycling and accelerating IgG degradation [195, 196]. Compared to full-length IgG, the YTE-KF construct showed stronger binding to membrane-associated FcRn and superior inhibition of FcRn-mediated IgG recycling in cynomolgus monkeys [197]. The FDA has approved efgartigimod for the treatment of myasthenia gravis in 2021 and for chronic inflammatory demyelinating polyneuropathy in 2024.

#### Enhancing effector functions via Fc afucosylation

Over the past decade, Fc core afucosylation has been established as a critical modification for enhancing ADCC in therapeutic IgG1 mAbs. Specifically, removal of fucose from the Asn297-linked Fc glycan markedly increases binding affinity to FcγRIIIa, thereby potentiating natural killer (NK) cell-mediated cytotoxicity. Importantly, this effector function is central to the anti-tumor activity of many IgG1-based therapies [198]. Several strategies have been used to modulate fucose levels, including selection of natural cell lines with low fucose modification activity as well as modulation of enzyme functions in CHO cell lines; activities have been modulated for alpha-(1,6)-fucosyltransferase (FUT8), GDP mannose 4,6 dehydratase (GMD), GDPL-fucose synthase (FX), GDP-fucose transporter SCL35C1, b-1,4-N-acetylglucoseaminyl-transferase III (GnTIII) [199].

Obinutuzumab (Gazyva) is a humanized IgG1 mAb targeting CD20; this product was approved in 2013 for the treatment of chronic lymphocytic leukemia. It is produced in glycoengineered CHO cell lines with overexpression of GnTIII, yielding antibodies with approximately 15% core fucosylation and improved ADCC relative to rituximab. In contrast, rituximab is produced in conventional CHO cells and retains high levels of core fucosylation, which lead to lower affinity for FcγRIIIa and reduced ADCC activity. Mogamulizumab (Poteligeo) is a humanized IgG1 mAb targeting CCR4, which was approved in 2018 for mycosis fungoides or Sézary syndrome. It is produced in FUT8-knockout CHO cells as a highly afucosylated antibody with enhanced ADCC against CCR4-expressing malignant T cells [200]. The FUT8-knockout CHO cell line is part of the POTELLIGENT® technology platform developed by BioWa, Inc., which produces antibodies that are nearly completely afucosylated. These antibodies therefore cause potent NK cell-mediated cytotoxicity through enhanced FcγRIIIa interaction. Inebilizumab (Uplizna; Amgen) was approved in 2020 for treatment of neuromyelitis optica spectrum disorder (NMOSD), a rare autoimmune disorder. The antibody is an anti-CD19 humanized IgG1 antibody produced in FUT8-knockout CHO cell line [198]. Benralizumab (Fasenra) was approved in 2024 for eosinophilic granulomatosis with polyangiitis and other eosinophilic disorders; this antibody is an anti-IL-5Rα humanized IgG1 antibody produced in in FUT8-knockout CHO cell line [198]. Margetuximab (Margenza), approved in 2020 for the treatment of HER2-positive metastatic breast cancer, is a chimeric IgG1 mAb targeting HER2. It is produced in FUT8-knockout CHO cells to achieve afucosylation and is Fc-engineered to include five amino acid substitutions (L235V/F243L/R292P/Y300L/P396L) that enhance binding-induced immune cell activation via FcγRIIIa while reducing affinity for the inhibitory FcγRIIb (CD32B) [201]. These modifications collectively enhance ADCC relative to trastuzumab, particularly in patients with low-affinity FcγRIIIa allotypes [202]. Belantamab mafodotin (Blenrep) was initially approved in 2020 for relapsed or refractory multiple myeloma and is an ADC targeting B-cell maturation antigen. Its IgG1 backbone is afucosylated through production in FUT8-knockout CHO cells to enhance ADCC [203]. Although the antibody was withdrawn in 2023, it remains under BLA review in 2025. Amivantamab (Rybrevant) was approved in 2021 for the treatment of NSCLC harboring EGFR exon 20 insertion mutations; this antibody is a humanized bispecific IgG1 mAb targeting both EGFR and MET receptors. It is produced in glycoengineered CHO cells with reduced fucosylation activity [204]. Ublituximab (Briumvi), approved in 2022 for the treatment of relapsing forms of multiple sclerosis, is a glycoengineered chimeric anti-CD20 IgG1 mAb. It is produced in YB2/0 cells and exhibits approximately 24% core fucosylation [198].

Methods have also been developed to create homogeneous antibodies featuring a specific, optimal glycan structure at the Fc region Asn297 position [205]. This structure is defined as a biantennary N-glycan capped with two terminal alpha-2,6-linked sialic acids (α2,6-SCT) and is recognized as a common optimal form. The key advantage of the structure is a significant enhancement of antibody effector functions like ADCC and CDC [206, 207]. The functions are primarily linked to increased binding affinity and activation of Fc receptors (especially FcγRIIIa and FcγRIIa) by α2,6-SCT. The structure is generated through both in vitro enzymatic glycan remodeling and cell-based glycan engineering, resulting in homogeneous preparations with potentially improved safety and efficacy [208].

#### Attenuating effector functions for immune modulation

Therapeutic antibodies for autoimmunity and immune checkpoint blockade are often engineered to minimize ADCC/CDC while preserving antigen binding.

*IgG4 subclass:* Human IgG4 and IgG2 subclasses are often considered desirable due to their inherently low affinity for Fcγ receptors (FcγRs) and complement component C1q. Modern IgG4 backbones incorporate the S228P hinge-stabilizing mutation to prevent Fab-arm exchange and preserve structural integrity. Antibodies built on this scaffold exhibit minimal FcγR engagement, which thereby attenuates ADCC and CDC [209–211]. Nivolumab (Opdivo) and pembrolizumab (Keytruda) are IgG4 mAbs targeting PD-1; each incorporates the S228P mutation. These antibodies were approved in 2014, and both rely on checkpoint blockade as their primary mechanism of action, with negligible Fc-mediated cytotoxicity [212, 213]. Similarly, cemiplimab-rwlc (Libtayo), a human IgG4 antibody against PD-1 containing the S228P mutation, was approved in 2018 for the treatment of cutaneous squamous cell carcinoma, basal cell carcinoma, and NSCLC [214, 215].

*N297A variant:* Atezolizumab (Tecentriq) is a humanized IgG1 mAb targeting PD-L1, which was first approved by the FDA in 2016 for advanced urothelial carcinoma. Later the indication was extended to include NSCLC. This antibody incorporates the N297A point mutation in the Fc region, which eliminates a conserved N-linked glycosylation site and abrogates binding to Fcγ receptors and C1q. This Fc-silent architecture effectively abolishes ADCC and CDC, minimizing off-target immune activation. The design preserves potent inhibition of the PD-1/PD-L1 axis, enabling reactivation of cytotoxic T cell responses and anti-tumor immunity [216, 217].

*LALA variant:* Etesevimab is a human IgG1 mAb targeting the RBD of SARS-CoV-2. It was developed in combination with bamlanivimab for the treatment of COVID-19 and received an EUA from the FDA from February 2021 to January 2022. Etesevimab incorporates LALA mutations (L234A and L235A) in the Fc region to attenuate its binding to Fcγ receptors. This Fc-engineering strategy significantly reduces Fc-mediated effector functions, presumably mitigating the risks of ADE and Fc-driven inflammation [218].

*FES variant:* Durvalumab (Imfinzi) is a human IgG1κ mAb targeting PD-L1, which was first approved in 2017 for urothelial carcinoma. Subsequent approvals were granted for NSCLC and other malignancies. To minimize Fc-mediated effector functions while maintaining its immune checkpoint blockade activity, durvalumab incorporates a triple amino acid substitution (L234F, L235E and P331S; FES), within the CH2 domain. These mutations abrogate binding to activating FcγRs and C1q, significantly reducing ADCC and CDC. This Fc-silent configuration reduces the risk of off-target immune activation and is particularly advantageous for long-term immunotherapy [201]. Anifrolumab (Saphnelo) is a fully human IgG1 targeting type I interferon receptor subunit 1 (IFNAR1) approved in 2021 for moderate to severe systemic lupus erythematosus (SLE). Like durvalumab, it incorporates the FES mutations to minimize interactions with FcγRs and C1q, in line with its intended immunomodulatory mechanism of action [219].

## New antibody-based modalities

### Antibody drug conjugates (ADCs)

In 1908, Paul Ehrlich proposed the concept of a "magic bullet" [220]. This strategy requires precise delivery of a drug to disease-causing cells in order to maximize therapeutic efficacy while minimizing off-target toxicity. ADCs embody the magic bullet concept by integrating targeted therapy with controlled drug distribution. Structurally, ADCs consist of three key components: an antibody, a linker and a payload [38]. Modified linkers are used to bridge antibodies and drugs, enabling the effective targeting of therapeutic payloads to antigen-bearing cells. Upon binding to surface antigens on cancer cells, ADCs are internalized into the cytoplasm via endocytosis. This process involves the formation of endosomes, which subsequently fuse with lysosomes. Inside the endosomes or lysosomes, intracellular enzymes cleave the drug payload from the ADCs, converting the payload into active free form drug that kills the target cells. Certain payloads may also exert a bystander killing effect in which drug released from dead target cells will impact neighboring cancer cells, further enhancing the therapeutic efficacy [38, 221].

#### Tumor-associated antigen-targeting ADCs

Advancements in mAb technology and large-scale manufacturing have significantly accelerated the progress of ADC development. As the targeting moiety in ADCs, the selection and development of targeting antibodies is undeniably crucial. The process begins with identifying a suitable tumor-specific/associated antigen for antibody development. Given the mechanism of ADCs, an ideal target antigen must possess certain characteristics, including reasonable tumor specificity, a non-secreted membrane-bound protein form, and the capacity for efficient internalization. Currently, the top five targets for ADCs under preclinical and clinical investigation are HER2, TROP2, CLDN18.2, B7-H3 and FRα. Each of these targets is promising for enhancing the precision and efficacy of ADC therapies [222]. Many ADC targets have been extensively studied for their roles in carcinogenesis. When developing antibodies for ADCs, functions such as specificity, affinity and immunogenicity are paramount. Regarding immunogenicity, antibody humanization technology has significantly enhanced the safety and therapeutic potential of ADC treatments. Furthermore, antibodies must exhibit sufficient pharmacokinetic properties after payload conjugation to ensure effective circulation, stability and target engagement. These considerations are critical in the early development process and require thorough evaluation to achieve maximum efficacy and minimal toxicity of an ADC product.

#### Linker types for ADCs

Researchers have become increasingly focused on ADC linker development to address critical challenges, such as improving the stability of the complex during delivery, achieving homogeneous ADC synthesis, and ensuring precise cleavage upon entry into target cells. ADC linkers are broadly categorized as non-cleavable and cleavable types. Notably, all FDA-approved ADCs, except trastuzumab emtansine (Kadcyla), utilize cleavable linkers [38, 221, 223], which generally confer low off-target toxicity due to the activation of drug release only under specific environmental conditions. The mechanisms of linker cleavage include chemical reduction, proteolysis, pH sensitivity and induction by radiation [224, 225]. One study reported that the most commonly used ADC linkers are valine-citrulline, Gly-Gly-Phe-Gly (GGFG), valine-alanine, SMCC, and beta-glucuronide [222]. Among these linkers, valine-citrulline, valine-alanine and GGFG are cleaved by cathepsin B [226], a lysosomal cysteine protease highly expressed in tumor cells. This characteristic enhances the efficiency of payload release, further improving ADC therapeutic efficacy.

#### Payload selection for ADCs

Equally important to the antibody and linker, the payload is a critical component of ADCs. Current ADC payloads primarily fall into three categories: microtubule inhibitors (auristatin and maytansinoid), topoisomerase I inhibitors (camptothecin derivatives), and DNA synthesis inhibitors (PBD dimers). Among FDA-approved ADCs, commonly utilized payloads include: derivative of auristatin (MMAE), derivatives of maytansinoid (DM1 and DM4), derivatives of camptothecin (SN-38 and DXd) and DNA alkylators (calicheamicin and PBD) [221, 222]. In early ADCs, microtubule inhibitors were commonly used as payloads for several FDA-approved molecules. However, a growing number of ADCs with microtubule inhibitors have been discontinued in clinical trials, often due to challenges such as limited efficacy or toxicity concerns. In more recent years, camptothecin derivatives have emerged as preferred payloads, offering a mechanism of action of topoisomerase I inhibition, which disrupts DNA replication and induces tumor cell death. This shift in preferred payload has led to a significant increase in ADC candidates utilizing camptothecin, with over 100 such experimental ADCs currently advancing through clinical trials [224]. The success of FDA-approved ADCs such as trastuzumab deruxtecan (DXd), sacituzumab govitecan (SN-38), and datopotamab deruxtecan (DXd) has significantly elevated expectations for camptothecin-based payloads.

#### Conjugation methods

The drug-to-antibody ratio (DAR) is a critical parameter for ADCs. Maintaining a homogeneous DAR across ADC batches is essential to ensure consistent chemical properties and predictable clinical performance. However, the choice of conjugation method significantly impacts the DAR profile. Two categories of ADC conjugation can be defined, random conjugation and site-specific conjugation [227]. Random ADC conjugation primarily occurs at lysine or cysteine residues within the IgG structure, leading to uncontrolled attachment sites and heterogeneous DARs. This heterogeneity can affect antibody stability, binding affinity and overall therapeutic efficacy. Random conjugation methods are more commonly employed in the early stages of ADC development and screening. In contrast, site-specific conjugation enables precise attachment of the payload, generating homogeneous ADCs with a well-defined DAR. This approach improves batch-to-batch consistency, enhances stability, and facilitates the optimization of the therapeutic window.

Common site-specific conjugation strategies include glycan modification, cysteine engineering, non-natural amino acid modification, and enzymatic conjugation. A particularly significant site for ADC conjugation is N297 in the Fc domain [228]. This site is not only the primary site of antibody glycosylation but also plays a crucial role in antibody functionality. Glycosylation at N297 stabilizes the antibody structure while enhancing ADCC and CDC, both of which contribute to immune-mediated tumor clearance. Due to the presence of only two N297 glycosylation sites in an IgG molecule, the DAR can be precisely controlled by utilizing the sugar chains at N297 as conjugation points. This control makes glycan modification at N297 a popular approach for site-specific ADC conjugation, despite its technical complexity. Recently, several companies have developed mature ADC platforms utilizing N297 glycosylation for conjugation, including GlycoConnect (Synaffix), SMARTag (Catalent), CHOptimax (CHO PHARMA) and GlycOBI (OBI Pharma) [229–233].

#### FDA-approved ADCs

**Fig. 4:**
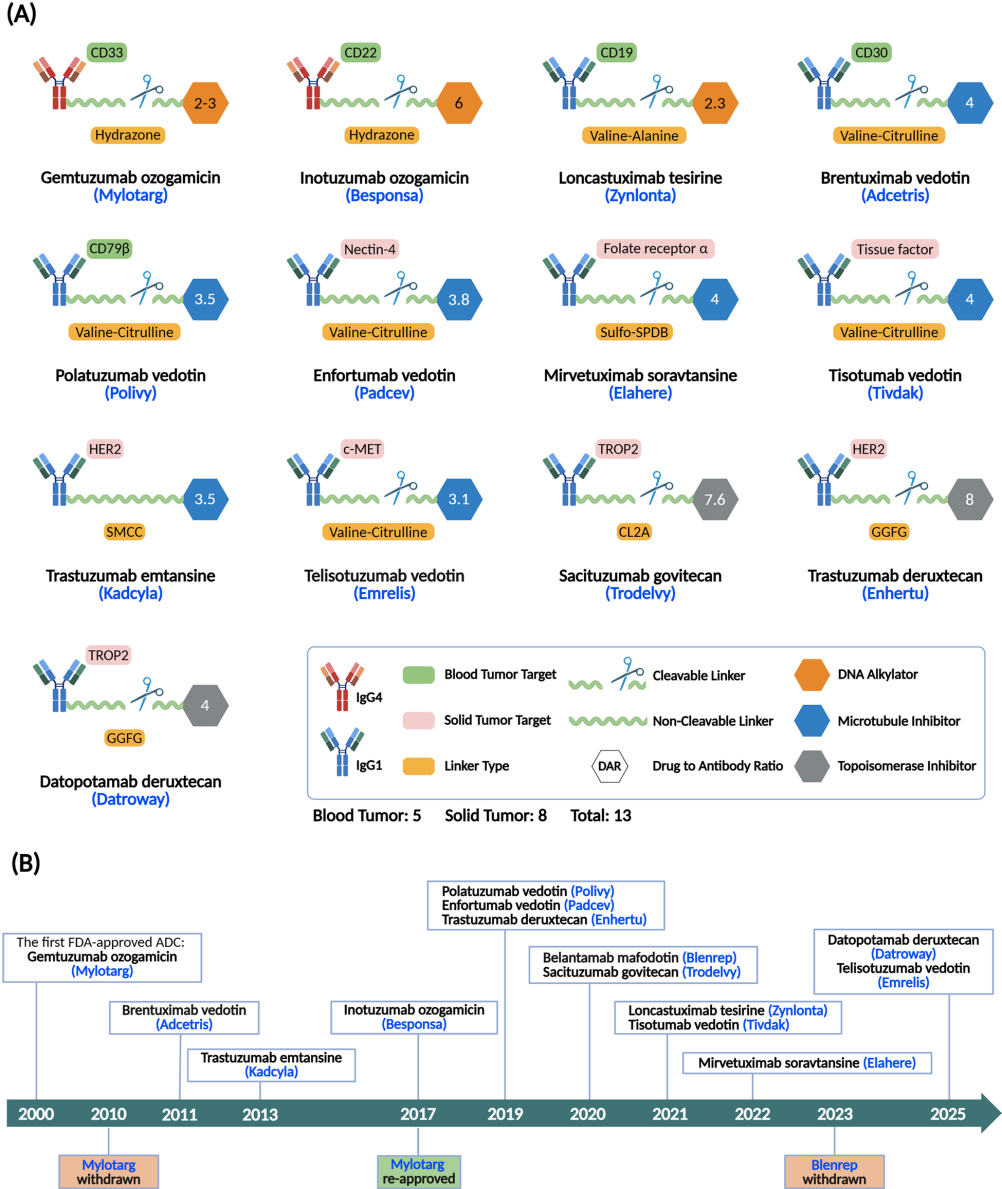
FDA-approved ADCs. **A** Schematic shows 13 FDA-approved ADCs on the market, detailing target tumor antigen, antibody format, linker type, drug payload, and drug-to-antibody ratio (DAR). **B** Timeline illustrates FDA-approved ADCs from 2000 to August 2025

As of now, 13 ADCs have been approved by the FDA for clinical use. The key characteristics of these molecules are summarized in Table 4, and their structural representations are shown in Fig. 4A. Among the approved ADCs, five are designed for the treatment of hematological cancers, while eight target solid tumors. Gemtuzumab ozogamicin (Mylotarg) was the first FDA-approved ADC. The drug was launched in 2000, withdrawn in 2010, and subsequently re-launched in 2017 (Fig. 4B). The history of FDA-approved ADCs highlights significant advancements in ADC-related technologies, including antibody engineering, cleavable linker design, payload selection, and conjugation strategies. These innovations have greatly improved the therapeutic potency and specificity of ADCs. For example, both Kadcyla and Enhertu utilize the same antibody, trastuzumab, to target HER2-expressing cancer cells. However, clinical data indicate that Enhertu demonstrates significantly improved patient survival outcomes compared to Kadcyla [234]. Comparing the design characteristics of Enhertu and Kadcyla, a key distinction lies in the linker regions. Enhertu employs a cleavable linker, which facilitates efficient payload release and enhances the bystander effect, allowing cytotoxic molecules to affect neighboring tumor cells. In contrast, Kadcyla utilizes a non-cleavable linker, restricting payload activity to directly targeted cells. Additionally, Enhertu is conjugated with eight molecules of camptothecin, whereas Kadcyla carries only 3.5 molecules of DM1, resulting in a higher DAR for Enhertu. These technological advancements in linker design, payload selection and DAR likely contribute to Enhertu’s superior efficacy in clinical outcomes compared to Kadcyla.

**Table 4:** FDA-approved ADCs on the market

No	Ab name	Target	Indication	Linker	Payload	Average DAR	U.S. approval	Global Sales in 2024 (millions USD)
1	Gemtuzumab ozogamicin (Mylotarg)	CD33	Acute myeloid leukemia	Cleavable (Hydrazone)	Calicheamicin	2–3	2000^#^; 2017	N/A
2	Brentuximab vedotin (Adcetris)	CD30	Hodgkin lymphoma, systemic anaplastic large cell lymphoma	Cleavable (Valine-Citrulline)	MMAE	4	2011	1,865
3	Trastuzumab emtansine (Kadcyla)	HER2	Breast cancer	Non-cleavable (SMCC)	DM1	3.5	2013	2,493
4	Inotuzumab ozogamicin (Besponsa)	CD22	Acute lymphoblastic leukemia	Cleavable (Hydrazone)	Calicheamicin	6	2017	N/A
5	Enfortumab vedotin (Padcev)	Nectin-4	Urothelial cancer	Cleavable (Valine-Citrulline)	MMAE	3.8	2019	2,193
6	Polatuzumab vedotin (Polivy)	CD79β	Diffuse large B-cell lymphoma	Cleavable (Valine-Citrulline)	MMAE	3.5	2019	1,573
7	Trastuzumab deruxtecan (Enhertu)	HER2	HER2 + metastatic breast cancer	Cleavable (tetrapeptide-based, GGFG)	DXd	8	2019	3,352
8	Sacituzumab govitecan (Trodelvy)	TROP2	Triple-neg. breast cancer	Cleavable (CL2A)	SN38	7.6	2020	1,315
9	Loncastuximab tesirine (Zynlonta)	CD19	Diffuse large B-cell lymphoma	Cleavable (Valine-Alanine)	PBD (SG3199)	2.3	2021	80
10	Tisotumab vedotin (Tivdak)	Tissue factor	Cervical cancer	Cleavable (Valine-Citrulline)	MMAE	4	2021	131
11	Mirvetuximab soravtansine (Elahere)	Folate receptor	Ovarian cancer	Cleavable (Sulfo-SPDB)	DM4	3.4	2022	479
12	Datopotamab deruxtecan (Datroway)	TROP2	NSCLC	Cleavable (tetrapeptide-based, GGFG)	DXd	4	2025	N/A
13	Telisotuzumab vedotin (Emrelis)	c-MET	NSCLC	Cleavable (Valine-Citrulline)	MMAE	3.1	2025	N/A

SMCC, succinimidyl-4-(N-maleimidomethyl cyclohexane)-1-carboxylate; GGFG, Glycine-Glycine-Phenylalanine-Glycine; sulfo-SPDB, 1-(2, 5-Dioxopyrrolidin-1-yloxy)-1-oxo-4-(pyridin-2-yldisulfanyl) butane-2-sulfonic acid; MMAE, Monomethyl auristatin E; DM1, N^2^'-Deacetyl-N^2^'-(3-mercapto-1-oxopropyl)-maytansine; DXd, Deruxtecan; SN38, 7-Ethyl-10-hydroxycamptothecin; PBD, Pyrrolobenzodiazepine; DM4, N^2^'-Deacetyl-N^2^'-(4-mercapto-4-methyl-1-oxopentyl)-maytansine

^#^Mylotarg was first approved in 2000, withdrawn in 2010, and re-approved in 2017

The growing success of FDA-approved ADCs has fueled major optimism in the field, with around 300 ADC candidates currently in clinical trials. Many of these clinical-stage ADC candidates show strong potential for future approval. For example, sacituzumab tirumotecan (sac-TMT) is designed for treatment of metastatic triple-negative breast cancer and has demonstrated encouraging results in phase III trials; the drug is expected to gain FDA approval in 2025 [235]. Although ADCs represent a promising therapeutic modality, several critical challenges still need to be addressed to maximize their clinical success, including stability of ADCs in delivery, drug resistance, and off-target effects [221, 224]. All of these obstacles will require technical advancements to improve the efficacy and safety of ADCs.

### Bispecific antibodies (bsAbs)

BsAbs are defined by their ability to simultaneously bind different antigens or distinct epitopes on the same antigen. Compared to traditional therapeutic antibodies, bsAbs offer several advantages and exhibit significant potential for development. For instance, the dual-targeting capability of bsAbs can enhance treatment efficacy, particularly in complex diseases like cancer, where multiple pathways may contribute to the disease. Additionally, targeting multiple antigens or pathways with bsAbs can help to reduce the occurrence of drug resistance [236].

In recent years, bsAbs have been developed for the treatment of various diseases, with a significant proportion (approximately 85%) being cancer therapies. Beyond oncology applications, bsAbs are also used to treat hematologic conditions (e.g., hemophilia), ophthalmic diseases (e.g., macular degeneration), and infectious diseases (e.g., rabies, COVID-19 and AIDS). Among bsAbs used for cancer therapy, the majority (~ 72%) are designed to treat solid tumors, while the remaining (~ 28%) target hematological malignancies. To date, 178 bsAbs have entered clinical trials and 15 of these have been approved by the FDA (Table 5).

**Table 5:** FDA-approved bispecific antibodies on the market

No	Ab name	Target	Indication	Format	U.S. Approval	Global Sales in 2024 (millions USD)
1	Blinatumomab (Blincyto)	CD19, CD3	Acute lymphoblastic leukemia	BiTE (Tandem scFv)	2014	1,302
2	Emicizumab (Hemlibra)	Factor IXa, Factor X	Hemophilia A	ART-Ig	2017	5,012
3	Amivantamab (Rybrevant)	EGFR, c-MET	NSCLC w/EGFR exon 20 insertion mutations	DuoBody [controlled Fab-arm exchange (cFAE)]	2021	1,200
4	Faricimab (Vabysmo)	VEGF-A, Ang-2	Neovascular age-related macular degeneration, diabetic macular edema	CrossMab [Knobs-into-holes (KiH)]	2022	4,301
5	Mosunetuzumab (Lunsumio)	CD20, CD3	Follicular lymphoma	IgG-like [Knobs-into-holes (KiH)]	2022	79
6	Tebentafusp (Kimmtrak)	gp100, CD3	Metastatic uveal melanoma	ImmTAC	2022	310
7	Teclistamab (Tecvayli)	BCMA, CD3	Multiple myeloma	DuoBody [controlled Fab-arm exchange (cFAE)]	2022	549
8	Elranatamab (Elrexfio)	BCMA, CD3	Multiple myeloma	Hinge mutation (IgG2a-like)	2023	118
9	Epcoritamab (Epkinly, Tepkinly)	CD20, CD3	Diffuse large B-cell lymphoma	DuoBody [controlled Fab-arm exchange (cFAE)]	2023	281
10	Glofitamab (Columvi)	CD20, CD3	Diffuse large B-cell lymphoma	CrossMab [Knobs-into-holes (KiH)] '2 + 1'	2023	191
11	Talquetamab (Talvey)	GPCR5D, CD3	Multiple myeloma	DuoBody [controlled Fab-arm exchange (cFAE)]	2023	NA
12	Tarlatamab (Imdelltra)	DLL3, CD3	Small cell lung cancer	HLE-BiTE	2024	92
13	Zanidatamab (Ziihera)	HER2, HER2 (biparatopic)	Biliary tract cancers	scFv-Fab-Fc [Azymetric Fc platform]	2024	1
14	Zenocutuzumab (Bizengri)	HER2, HER3	Neuregulin 1 fusion (NRG1 +) NSCLC and NRG1 + pancreatic cancer	DEKK	2024	NA
15	Linvoseltamab (Lynozyfic)	BCMA, CD3	Multiple myeloma	VelociBi	2025	NA

#### Formats of bsAbs

To achieve optimal therapeutic effects, bsAbs are designed with structures and formats tailored to specific therapeutic applications. An increasing number of technologies are being utilized to generate desired bsAb formats. Broadly speaking, bsAbs can be divided into two main classes, IgG-like and non-IgG-like [237]. IgG-like bsAbs have larger molecular sizes and contain Fc fragments, which enhance stability and prolong serum half-life. Additionally, the Fc region of IgG-like bsAbs can mediate various immune functions, such as ADCC, antibody-dependent cellular phagocytosis (ADCP), and CDC. In contrast, non-IgG-like bsAbs are smaller and more structurally flexible, while exhibiting better tissue penetration and lower immunogenicity. However, the reduced stability of non-IgG-like bsAbs may result in a shorter half-life, necessitating more frequent dosing. To address this issue, bsAbs that do not include an Fc portion may be fused with human serum albumin (HSA), polyethylene glycol (PEG) or other molecules to extend their half-life.

IgG-like bsAbs can be further divided into two sub-categories: asymmetric and symmetric. Compared to symmetric bsAbs, the production of asymmetric bsAbs poses greater challenges in ensuring correct assembly and folding. To address these challenges, strategies have been developed to improve heterodimerization and prevent mispairing during production, including knobs-into-holes (KiH), DEKK, asymmetric reengineering technology-Ig (ART-Ig), charge repulsion improved bispecific (CRIB), strand-exchange engineered domain (SEED), bispecific engagement by antibodies based on the T cell receptor (BEAT), DuoBody, CrossMAb, and Wuxibody. The purpose of these technologies is mainly to prevent mispairing of the heavy chains, while some strategies also prevent mispairing of light chains [238].

For IgG antibodies, the Fc portion plays critical roles in extending half-life, mediating immune effects, and facilitating purification. In the design of bsAbs, Fc engineering technologies can be employed to maintain correct antibody structures and enhance therapeutic efficacy. The methods described above (e.g., KiH, SEED and ART-Ig) involve introducing mutations into the Fc region to promote the formation of heterodimeric bsAbs. Furthermore, modifications to the Fc region can be introduced to either enhance or attenuate immune responses. These modifications include improving interactions with FcRn to prolong serum half-life or reduce immunogenicity to minimize adverse effects.

#### Mechanisms of action for bsAbs

**Fig. 5:**
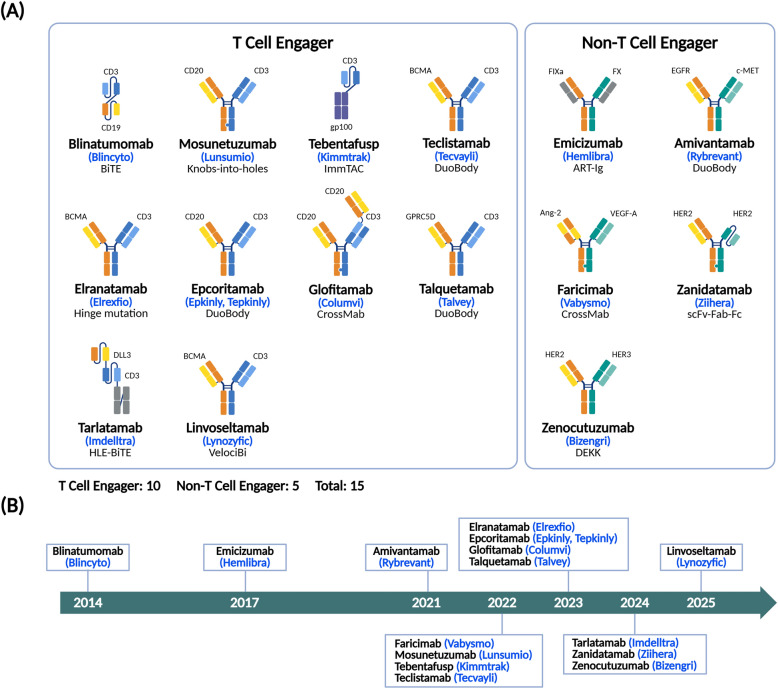
FDA-approved bispecific antibodies. **A** Structures of 15 bsAbs approved by the FDA (as of August 2025) are shown, comprising 10 T cell engagers and 5 non-T cell engagers. Among the 15 bsAbs, three are classified as non-IgG-like formats, while the remaining 12 exhibit IgG-like formats. Within the non-IgG-like category, blinatumomab is a bispecific T cell engager (BiTE) composed of tandem scFvs. Tebentafusp is an ImmTAC molecule, which features a high-affinity, soluble TCR domain fused to an anti-CD3 scFv. Tarlatamab consists of two scFvs and incorporates an Fc domain to extend its half-life. In the IgG-like formats, various heterodimerization strategies are employed, including Knobs-into-holes (e.g., mosunetuzumab); DuoBody (e.g., amivantamab, teclistamab, epcoritamab, and talquetamab); CrossMab, which incorporates Knobs-into-holes technology (e.g., faricimab and glofitamab); ART-Ig (e.g., emicizumab); hinge mutation (e.g., elranatamab); DEKK (e.g., zenocutuzumab); and VelociBi (e.g., linvoseltamab). **B** Timeline of FDA approvals for bsAbs. Blinatumomab was the first bsAb approved by the FDA. The number of bsAbs approved by the FDA has increased substantially since 2022

BsAbs possess the capacity to simultaneously bind two distinct epitopes, so these molecules exhibit mechanisms of action that are inherently more complex than those of conventional monospecific antibodies. The mechanisms of bsAb action can be classified as several distinct modalities, with complexity arising from the interplay between dual antigen binding and the spatial distributions of target antigens. The principal mechanisms include the following. (1) Immune cell bridging: In this modality, one arm of the bsAb binds a tumor-associated antigen (TAA), while the other engages an immune cell surface marker (e.g., CD3 on T cells). This dual engagement facilitates the formation of an “immune synapse,” leading to T cell activation and subsequent tumor cell lysis. For instance, blinatumomab (CD3 × CD19) and mosunetuzumab (CD20 × CD3) each redirect T cells to malignant B cells by concurrently binding TAAs and CD3, thereby promoting cytotoxic activity [239, 240]. Among the 15 FDA-approved bsAbs, ten are classified within this category (Fig. 5); (2) Dual signaling modulation: BsAbs can modulate intracellular signaling pathways by binding two receptors either on the same cell (cis) or on different cells (trans), resulting in synergistic effects. Amivantamab (EGFR × c-MET) concurrently inhibits EGFR and mesenchymal-epithelial transition factor (c-MET) signaling, thereby overcoming resistance to targeted therapies in NSCLC [241]; (3) Forced protein complexation: In this approach, the bsAb mediates formation of a complex between two proteins, effectively substituting for a missing or deficient functional molecule. Emicizumab (FIXa × FX) exemplifies this modality by mimicking coagulation factor VIII activity and facilitating factor X activation in patients with hemophilia A [242]; (4) Biological barrier transcytosis: Certain bsAbs can traverse physiological barriers by exploiting receptor-mediated transport. For example, anti-transferrin receptor (TfR) × BACE1 bsAbs leverage TfR-mediated transcytosis to deliver therapeutic antibodies across the blood-brain barrier, thereby reducing β-amyloid (Aβ) accumulation in the treatment of Alzheimer’s disease [243]; (5) Immune checkpoint synergy: This modality involves concurrent targeting of multiple immune checkpoint molecules (e.g., PD-1 and CTLA-4) in order to attenuate the immunosuppressive tumor microenvironment. Compared to combination therapy with mAbs, bsAbs may exhibit enhanced target specificity with reduced systemic toxicity [244].

#### Challenges related to bsAbs

BsAbs exhibit significant therapeutic advantages and developmental potential; however, several challenges must be addressed to optimize their clinical application. (1) Production challenges: The structural complexity of bsAbs introduces substantial manufacturing hurdles, such as low yield, impurities, chain mispairing, misfolding and aberrant glycosylation [245]. Even when target products are successfully synthesized, aggregation and instability during storage or delivery remain critical concerns. Advanced engineering platforms have improved heavy-light chain assembly accuracy and reduced levels of mispaired species. (2) Pharmacokinetic limitations: BsAbs display distinct profiles compared to conventional mAbs, influenced by factors such as molecular size, physicochemical properties, Fc modifications, and antigen-binding valency [236]. While smaller bsAb formats offer enhanced tissue penetration, these drugs are subject to rapid systemic clearance, which necessitates frequent dosing. Conversely, larger molecules demonstrate prolonged half-lives with limited biodistribution into target tissues, potentially compromising therapeutic efficacy. To address these limitations, half-life extension technologies, such as the addition of albumin-binding domains or Fc engineering, have been developed. These technologies to enhance stability may be utilized alongside high-throughput screening and computational modeling to optimize antigen affinity, specificity and structural stability. (3) Safety risks: BsAbs may trigger safety concerns such as cytokine release syndrome (CRS), off-target cytotoxicity, or undesirable immune activation [238]. Fc-mediated interactions with immune cells can amplify inflammatory responses, while cross-reactivity with non-target tissues increases toxicity risks. To mitigate these issues, conditional activation mechanisms have been explored to restrict antibody functionality to specific microenvironments, and immunosuppressive domains or deimmunization approaches have been employed to reduce CRS and immunogenicity. These advancements underscore the potential for integrated approaches of protein engineering, bioprocess optimization, and preclinical validation to overcome existing barriers in bsAb development.

#### Innovations in bsAbs

Advanced AI tools are being applied across various fields, including antibody discovery. From the preliminary identification of molecular targets to the design and optimization of sequences and structures, many different development steps can now be more efficiently accomplished using AI technology. Furthermore, AI-driven models have shown promising results in predicting binding affinity, immunogenicity and developability of bsAbs, significantly accelerating the drug discovery pipeline [246, 247]. By integrating large-scale biological datasets and advanced machine learning algorithms, AI offers new possibilities for identifying novel antibody formats and enhancing the precision of therapeutic design.

As bsAbs possess two distinct antigen-binding sites and exhibit complex mechanisms of action, the molecules exhibit pharmacokinetic profiles that are markedly different than monospecific antibodies. In the development of bsAbs, antigen-binding sites must be considered in pairs, and the molecular structure and format design should align with the intended therapeutic mechanism of action. Consequently, it is challenging to directly extrapolate existing data from monospecific antibodies to the development of bsAbs, necessitating the establishment of AI models specifically for bsAbs. One study utilized single-cell gene expression data from various cell types along with a curated list of bsAbs that are either approved or undergoing clinical trials as inputs to predict optimal target pairs for bsAb development. This approach demonstrably enhanced prediction accuracy [248]. As AI tools continue to evolve, their role in bsAb discovery is expected to expand, and the new tools are expected to provide innovative solutions to longstanding challenges in biopharmaceutical research, ultimately improving the efficiency and success rate of antibody-based therapeutic development.

Improvements to bsAb development are ongoing. The next generation of bsAbs includes various innovative concepts, such as bsAb-drug conjugates, prodrug-based bsAbs, and mRNA-LNP delivery systems for bsAbs [249]. Therefore, a major opportunity exists to develop novel molecules with improved efficacy, reduced toxicity, and broader applications. In particular, the integration of bsAbs with emerging delivery platforms, including nanoparticle-based and exosome-mediated systems, holds great promise for overcoming limitations of poor pharmacokinetics, immunogenicity, and restricted tumor penetration [250, 251]. Moreover, the ability to precisely control the spatial and temporal release of bsAbs through prodrug or stimuli-responsive designs can further enhance therapeutic outcomes while minimizing off-target effects [252]. Recent advances in synthetic biology and protein engineering have also enabled the generation of highly specific and customizable bsAbs tailored for various non-oncology disease indications, including autoimmune disorders, infectious diseases, and neurodegenerative conditions [253–255].

As the therapeutic landscape continues to evolve, the synergistic combination of bsAbs with other modalities, such as immune checkpoint inhibitors, CAR-T cells, and cancer vaccines, is expected to greatly expand the potential of immunotherapy [256, 257]. Additionally, ongoing efforts to optimize manufacturing processes, stability profiles, and cost-effectiveness will be crucial for facilitating clinical translation and commercial viability of next-generation bsAbs. Collectively, these innovations position bsAbs as a versatile and powerful class of therapeutics poised to address currently unmet medical needs.

### CAR-T cell therapy

CAR-T cells, often considered as living drugs, have gained enormous attention as a revolutionary personalized cancer immunotherapy. All approved CAR-T cell products are autologous ex vivo expanded T cells that have been genetically engineered to express synthetic receptors. There are also ‘off-the-shelf’ allogenic CAR-T cells under development. For these allogenic cells, T cells are collected from healthy donors and genetically engineered to specifically express CARs and evade the host immune system before infusion into the patient [258, 259]. Infused CAR-T cells recognize TAAs on the cancer cell surface to stimulate T cell-mediated cytotoxicity [260, 261]. The intracellular region of the CAR is activated by antigen binding and causes release of cytotoxic components through an immune synapse in order to cause cancer cell death [262]. Current CARs are composed of four main components, including the antigen-recognizing extracellular moiety (often derived from the variable region of an antibody), a hinge component that connects the extracellular and the trans-membrane region, a trans-membrane domain, and intracellular region that activates the T cells. The complex intracellular domain comprises various signaling transduction components that vary by the generation of CAR. For instance, the intracellular component in first generation CARs consists only of a CD3ζ domain to drive activation of T cells; first-generation CAR-T cells thus generally exhibit low cytotoxicity and antitumor efficacy [262, 263]. The intracellular components of second-generation CARs contain a CD28 co-stimulatory domain along with the CD3ζ domain, which markedly enhances T cell proliferation potential [260, 261]. The third and fourth generation CARs are further upgraded with the incorporation of additional signaling domains such as 4-1BB and interleukin (IL)-12 inducer domains with CD3ζ domain in complex intracellular constructs. Insertion of additional constructs has even led to the generation of a new cell type called T cell redirected for antigen-unrestricted cytokine-initiated killing (TRUCKs); these TRUCKs exhibit both cytotoxic and cytokine-releasing activities to kill targeted cancer cells [264, 265]. The most recent, fifth generation of CAR-T cells express CARs with an intracellular structure resembling that of the second generation CARs, but with incorporation of an additional truncated cytoplasmic IL-2 receptor β-chain domain; thus, the intracellular domain of a fifth-generation CAR is a complex of CD3ζ, CD28, IL-2 and 4-1BB domains [261, 266]. Another example of fifth-generation CAR design may be to include a truncated cytoplasmic receptor and transcription factor (STAT3/5)-binding motif into a second-generation design. Such complex CAR constructs have reported to activate anti-tumor immune responses and exhibit superior persistence in vivo [267]. The fifth-generation CAR constructs may also armor CAR-T cells with modulators or drug-dependent safety switches to improve the drug function and optimize therapeutic efficacy and safety. Nevertheless, limitations such as off-target toxicity and low-tumor efficacy still pose significant challenges for CAR-T cells, and these challenges must be overcome by technological advancements [265, 268].

#### Clinical applications of CAR-T cell therapy

Up to now, CAR-T cell therapy has only been used clinically to treat hematological malignancies. The FDA has approved seven CAR-T cell therapies for hematological cancers, five of which target CD19 and are used to treat B cell leukemias and lymphomas [269–271]. The two other FDA-approved CAR-T cell therapies are used for the treatment of multiple myeloma, and the cells are targeted against B cell maturation antigen (BCMA) [272, 273]. These FDA-approved CAR-T therapies can therefore be utilized against variety of hematological cancers (Table 6). In spite of the remarkable success of CAR-T cell therapies for treating refractory blood cancers, the technology has not yet been successfully applied to treat solid tumors. Reasons for this lack of clinical success against solid tumor targets are thought to be several-fold.

**Table 6:** FDA-approved CAR-T therapies

No	Drug name	Target	Indication	CAR Construct	U.S. Approval	Global Sales in 2024 (millions USD)
1	Tisagenlecleucel (KYMRIAH)	CD19	Relapsed and refractory B cell lymphoma	Anti-CD19-CD8α-4-1BB-CD3ζ	2017	443
2	Axicabtagene Ciloleucel (YESCARTA)	CD19	B cell lymphoma	Anti-CD19-CD28-CD3ζ	2017	1,570
3	Brexucabtagene Autoleucel (TECARTUS)	CD19	Relapsed and refractory B cell lymphoma	Anti-CD19-CD28-CD3ζ	2020	403
4	Lisocabtagene Maraleucel (BREYANZI)	CD19	B cell lymphoma	Anti-CD19-IgG4-CD28-4-1BB-CD3ζ	2021	747
5	Idecabtagene Vicleucel (ABECMA)	BCMA	Multiple myeloma	Anti-BMCA-CD8α-4-1BB-CD3ζ	2021	406
6	Ciltacabtagene Autoleuce (CARVYKTI)	BCMA	Multiple myeloma	Dual anti-BMCA-CD8α-4-1BB-CD3ζ	2022	963
7	Obecabtagene autoleucel (AUCATZYL)	CD19	Relapsed and refractory B-cell precursor acute lymphoblastic leukemia	Anti-CD19-CD8α-4-1BB-CD3ζ	2024	NA

#### Challenges related to CAR-T therapy for solid tumors

A major challenge in developing CAR-T cell therapies for solid tumors is the identification of appropriate tumor-associated targets due to high tumor heterogeneity. Although tumor heterogeneity exists in hematological malignancies, the targeted cancer cells invariably express CD19 or BCMA. In contrast, solid tumors display high intra-tumor variability and heterogeneity. In addition, expression of tumor-associated antigens on solid tumors may exhibit high variability within the same or different tumors, and the antigens may be expressed in essential non-tumor cells [274, 275]. Despite such challenges, significant efforts have been made to improve CAR-T therapies. In one strategy, multi-specific CAR-T therapies express CARs targeting two or more antigens; these CARs may be tandem CARs (TanCAR), bicistronic CARs or LoopCARs [276–278]. CAR-T therapy for the solid tumors must also exhibit sufficient tumor trafficking and successful infiltration of CAR-T cells into the tumor. In particular, the complex nature of the solid tumor stroma and abnormal vasculature impede efficient penetration and homing of CAR-T cells [274, 275, 279]. Consequently, new studies have sought to harness chemokines relevant to the tumor microenvironment (TME) in order to steer CAR-T cells into the solid tumors. Several chemokines are known to regulate immune cell trafficking and infiltration into tumors [279]. For instance, CXCR2-modified CAR-T cells have been generated to enhance trafficking of CAR-T cells in hepatocellular carcinoma [280]. Another major challenge relates to persistence and survival of CAR-T cells in the TME. Tumors are often enriched with immune-suppressive cell-types, such as tumor-associated macrophages (TAMs) or myeloid-derived suppressor cells (MDSCs), and they also may exhibit hypoxic conditions that impede CAR-T cell function [281]. Thus, recent studies have assessed TME-targeting approaches to enhance CAR-T cell activity. Such efforts include PD-1 gene editing, TGFβ-knockout and several other approaches to target various crucial TME factors [282–284].

In recent years several clinical trials have been conducted to assess CAR-T cells for the treatment of different solid cancers. Major indications have included triple-negative breast cancer, glioblastoma, lung, liver cancer, neuroblastoma, pancreatic cancer, gastrointestinal cancer, ovarian cancer and prostate cancer [261]. Some common CAR-T cell targets are Mucin 1 (MUC1), mesothelin, EGFR, CEA, CLDN18.2, GD2, PSCA, GPC-3and NKG2D [261]. A summary of several ongoing CAR-T therapy clinical trials for solid tumors is shown in Table 7. These clinical programs suggest the importance and potential utility of CAR-T therapy for treatment of solid tumors.

**Table 7:** CAR-T therapy candidates for solid tumors in clinical trials

No	Target	CAR Construct	Indications	Trial stage (trial number)
1	MUC-1	Anti-MUC1-CD28-CD3ζ	Breast Cancer	Phase 2 NCT05812326
2	GD2	Anti-GD2-CD28-4-1BB -CD3ζ	Neuroblastoma	Phase 1/2 NCT03373097
3	CLDN18.2	Humanized anti-CLDN18.2-CD8α-CD28- CD3ζ	Advanced gastric or gastroesophageal junction adenocarcinoma, Gastrointestinal cancer, Pancreatic cancer	Phase 1/2 NCT04581473 NCT04404595
4	CEA	Anti-CEA-CD8α-CD28-4-1BB-CD3ζ	Colorectal cancer, pancreatic cancer, NSCLC	Phase 1/2 NCT05736731
5	EGFR	Anti-EGFR-CAR	Lung, liver and stomach cancer	Phase 1/2 NCT02873390 NCT02862028
6	Mesothelin	Anti-mesothelin-4-1BB- CD3ζ	Lung Adenocarcinoma, ovarian cancer and pancreatic cancer	Phase 1 NCT03054298 NCT03323944 NCT05057715
7	EGFR	Anti-EGFRvIII-4-1BB- CD3ζ	Glioblastoma	Phase 1 NCT03726515
8	PSCA	Anti-PSCA-4-1BB/TCRzeta-CD19t	Prostate cancer	Phase 1 NCT03873805
9	GPC-3	Anti-GPC3-CD8-CD28- CD3ζ	Hepatocellular carcinoma	Phase 1 NCT02395250
10	NKG2D	NKG2DR-CD3ζ	Unresectable metastatic colorectal cancer	Phase 1 NCT03018405

#### Prospects for CAR-T therapy

CAR-T therapy has proven successful for treatment of hematological malignancies, but several limitations remain to be overcome. For instance, toxicity and long-term adverse effects still arise in a significant proportion of treated patients. Moreover, disease relapse may occur even after deep-minimal residual disease-negative remission [285]. As described above, developing effective CAR-T therapies for solid tumors has been a major challenge due to issues such as identification of appropriate TAAs. Notably, efforts are being made to overcome each of these barriers. For instance, numerous preclinical studies are being conducted to evaluate targeting of tumor-associated factors, such as fibroblast growth factor receptor 1 protein (FGFR1), platelet-derived growth factor receptor alpha (PDGFRA), and VEGF receptor 2 (VEGFR2) [275, 276, 282]. In recent preclinical studies, the potential for in vivo CAR-T therapy has been evaluated; this technology involves delivering specific CAR genes to T cells in the patient’s body using viral vectors or nanocarriers. Since the safety and efficacy of such novel therapeutics are a major concern for clinical application, regulatory bodies will require stringent quality checks prior to allowing clinical advancement [261]. Another major issue with ex vivo CAR-T therapy is the high cost of the therapy, which makes it unaffordable for many people. ‘Off-the-shelf’ allogenic strategies could be beneficial for lowering cost, but the technology is still not sufficiently mature for clinical implementation [259, 261]. Although many obstacles to clinical application of further CAR-T therapies remain, ongoing research efforts and industry investments are poised to maximize the potential of CAR-T therapy.

### mRNA-LNPs

Therapeutic antibodies have proven effective for the treatment of various diseases over the past few decades. However, the production costs for antibodies are high, and their clinical utility is often restricted by their short half-life [286, 287]. Another limitation is that targeted delivery of antibodies is challenging. To address these limitations, strategies have been devised to stimulate in vivo production of antibodies via mRNA-encoded molecules. This strategy minimizes the risk of immunogenicity from manufactured antibodies, as the desired antibodies are produced by the host cells [9, 288], offering a potentially safe and efficient means of overcoming many of the obstacles associated with traditional antibody therapies. In line with this concept, Tai et al., identified a mAb called 8-9D that neutralizes SARS-CoV-2 variants. Subsequently, they constructed 8-9D-encoding mRNA and encapsulated the mRNA in lung-selective lipid nanoparticles to enhance the neutralizing potential of the respective antibody. The study results demonstrated the feasibility of mRNA-based technology, highlighting the benefits of tissue targeting for the in vivo production of neutralizing antibodies over direct administration of manufactured neutralizing antibodies [57]. In another study, the authors delivered mRNAs encoding complex fusion proteins, SIRPα-Fc-CD40L and TIGIT-Fc-LIGHT, which require in vivo oligomerization to induce antitumor immunity. Intravenous administration of the designed mRNA-LNPs resulted in sustained in vivo production of functional hexametric proteins. This approach led to increased levels of the proteins (∼28- to 140-fold over 96 h) as compared with injected recombinant protein controls. Furthermore, Stadler et al. published preclinical data supporting the in situ generation of bsAbs using engineered in vitro transcribed (IVT) modified mRNAs termed RiboMABs. The designed mRNAs served as templates for production of bsAbs targeting CD3ε and CD19 on immune cells. Administration of just a few micrograms of RiboMAB-encoding mRNA was sufficient to induce rapid antibody production. Importantly, the in situ production of bsAbs via mRNA-LNP technology does not require case-by-case optimization, making the development process highly efficient. Thus, the approach could potentially enable rapid evaluation of different antibody modifications and conditions within a short period of time [289].

The clinical application of mAbs largely depends on achieving high serum antibody titers and extended half-lives to enable rapid and sustained activity. Several studies have demonstrated that encapsulation of mRNA in LNPs can be utilized to produce high levels of serum antibodies. Furthermore, it has been suggested that the LNP-mRNA platform offers superior therapeutic potential compared to traditional recombinant protein-based modalities [9, 290, 291]. However, antibody production via mRNA technology still faces two key limitations: (i) protein expression is dependent on the delivery system, such as the type of ionizable lipids or polymeric nanoparticles used; (ii) efficacy can vary depending on the route of administration. To enable successful in situ antibody generation in clinical settings, the remaining challenges associated with mRNA delivery and expression must be resolved through further technological advancements.

## Technological horizons for new antibody-based modalities

**Fig. 6:**
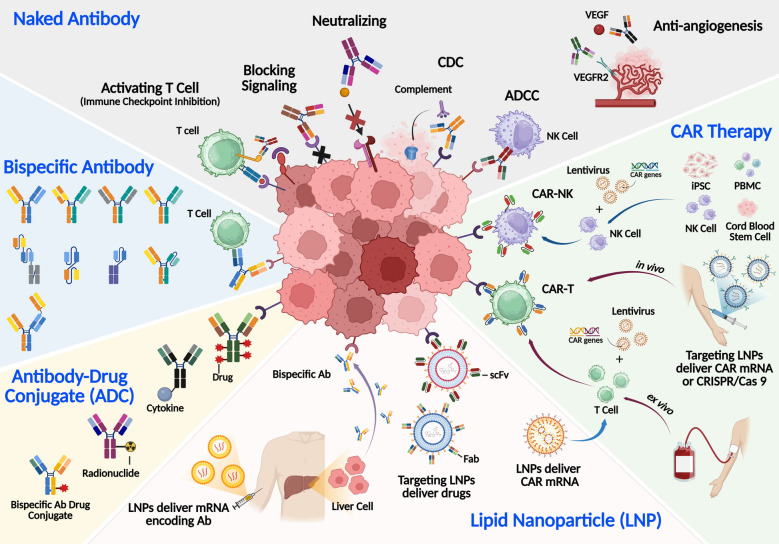
Mechanisms of antibody-based tumor therapy strategies. The central illustration represents a tumor microenvironment targeted by diverse antibody-based therapeutic approaches. Naked antibody: Naked antibodies mediate anti-tumor effects through multiple mechanisms: (1) antibody-dependent cellular cytotoxicity (ADCC), involving NK cells and macrophages; (2) complement-dependent cytotoxicity (CDC), triggering the complement cascade; (3) neutralization of tumor growth signals; (4) blockade of angiogenesis to inhibit tumor vascularization; (5) activation of T cells to enhance immune responses; and (6) inhibition of immune checkpoints (e.g., PD-1/PD-L1) to promote T cell and NK cell-mediated cytotoxicity. Bispecific antibody (bsAb): BsAbs, such as bispecific T-cell engagers (BiTEs), simultaneously bind TAAs and immune effector cells (e.g., T cells or NK cells), facilitating targeted cytotoxicity, often in combination with immune checkpoint inhibitors to amplify anti-tumor immunity. CAR-T therapies: CAR-T cells are generated by isolating T cells from patient peripheral blood, followed by genetic engineering for tumor-specific CAR expression. In contrast, CAR-NK cells are derived from induced pluripotent stem cells (iPSCs), peripheral blood mononuclear cells (PBMCs), NK cell lines, or umbilical cord blood (UCB). The cells are engineered with CAR genes to enhance tumor recognition and killing. Antibody-drug conjugate (ADC): ADCs consist of mAbs conjugated to cytotoxic payloads (e.g., cytokines or radionuclides), enabling precise delivery of the drug to tumor cells for targeted cytotoxicity. Lipid nanoparticle (LNP): (1) Targeting LNP: LNPs are conjugated with targeting ligands like scFvs or Fab fragments on their surface, enabling specific targeting of tumor sites and delivery of encapsulated anti-tumor drugs. Targeting LNPs also can deliver CAR-encoding mRNAs or CRISPR/cas9 for in vivo CAR-T and gene therapy. (2) LNP delivery of mRNA-encoding antibodies: LNPs encapsulate mRNAs encoding Abs or bsAbs. mRNA-LNPs administered via intravenous (iv) injection are taken up by hepatocytes, dendritic cells or other antigen-presenting cells. These cells translate the mRNA and release functional antibodies, effectively utilizing the human body as a bioreactor for antibody production, bypassing traditional protein drug manufacturing processes

Antibody-based therapeutics have become cornerstone treatments of human diseases. These drugs are mostly applied in the field of oncology but find usages in other diseases as well. Antibody-based therapeutic mechanisms of action are outlined in Fig. 6 for naked antibodies, bsAbs, CAR-T cells, ADCs, and LNP-based approaches. Future advancements in these therapeutic domains will hinge on the integration of AI, high-throughput sequencing technologies, immune checkpoint inhibitors, and innovative delivery systems. It is expected that further advancements will yield significant improvements in precision, efficacy and scalability of antibody-based treatments.

Recent advances in mRNA-LNP technology have allowed for targeted delivery of therapeutic proteins, such as cytokines, tumor suppressors and immunotoxins. For example, encapsulation of mRNAs encoding immunotoxins like *pseudomonas exotoxin* A (PE) within LNPs has been shown to induce tumor apoptosis and suppress growth in melanoma models [292]. Other groups have reported novel LNP designs that enable intratumoral delivery of bacterial enzyme mRNAs or redirect T cell immunity toward tumors, both resulting in significant tumor inhibition in preclinical models [293, 294]. To enhance cell-type specificity and reduce off-target effects, surface conjugation of targeting ligands, particularly antibody fragments such as scFvs or Fabs, has been extensively explored. Although still in early development, this approach has shown promise for targeting various organ systems. For instance, dendritic cells have been successfully targeted using DEC205 scFv-coated LNPs to enhance siRNA uptake [295], while lung-targeted delivery has been achieved with anti-PCAM-1 and anti-PV1 antibody-conjugated LNPs (anti-PCAM-1, > 25-fold increase in protein expression; anti-PV1, > 40-fold increase in mRNA delivery, as compared to non-targeted controls) [296, 297]. For targeting the central nervous system, LNPs functionalized with antibodies against VCAM-1 showed preferential accumulation in inflamed brain vasculature and effectively delivered thrombomodulin mRNA, alleviating TNF-α-mediated cerebrovascular edema [298].

In hematological applications, antibody-guided LNPs targeting c-kit enabled efficient gene editing in approximately 90% of hematopoietic stem and progenitor cells following a single systemic dose, with preserved stemness and multilineage potential [299]. Notably, Park and colleagues proposed a chemical-free strategy to functionalize LNPs by fusing apolipoprotein to the Fc domain of targeting antibodies, mimicking natural protein corona formation without altering LNP formulation. This approach enabled trastuzumab-bound mRNA-LNPs to selectively deliver p53 tumor suppressor mRNA to HER2-positive cancer cells, leading to complete tumor regression with no systemic toxicity [300]. Dietmair et al. developed molecules with bispecific scFvs (anti-PEG × anti-EGFR and anti-PEG × anti-PSMA) to direct mRNA-LNPs to breast cancer cells in xenografted mice [251].

Despite the success of CAR-T cell therapy in hematologic malignancies, several barriers still hinder its widespread application in solid tumors. These include on-target off-tumor toxicity, T cell exhaustion, and limited persistence [275]. Innovations in synthetic biology, such as sonogenetics, now enable remote and spatiotemporal control of CAR activation. For instance, the EchoBack-CAR-T cells include ultrasound-inducible promoters with positive feedback from CAR signaling to enhance the longevity and cytotoxicity of GD2-targeting CAR-T cells in glioblastoma models [301]. In addition, combining CAR-T therapy with immune checkpoint inhibitors, such as anti-PD-1/PD-L1 antibodies, can lead to synergistic effects via mitigation of T cell exhaustion and enhanced anti-tumor response [302–304].

To circumvent the costly and complex ex vivo CAR-T manufacturing process, in vivo CAR-T cell programming is gaining traction. Nobel laureate Dr. Jennifer A. Doudna’s team recently introduced an innovative approach for in vivo selective genome editing using antibody-targeted Cas9-enveloped delivery vehicles (Cas9-EDVs) [305]. By displaying scFvs against CD3, CD4 and CD28 on Cas9-EDVs, the team selectively generated CD19-targeting CAR-T cells from human T cells in the context of humanized mice. Thus, the Cas9-EDV approach represents a programmable and virus-free platform for precise cellular reprogramming.

Of late, mRNA-LNP technology has emerged as a powerful platform for in vivo CAR-T cell generation. For example, Rurik et al. employed CD5-targeted LNPs encapsulating anti-fibroblast activation protein (FAP) scFv-CAR mRNA to successfully reprogram T cells in vivo, mitigating fibrosis and restoring cardiac function in a mouse model of heart failure [306]. Similarly, Billingsley et al. used CD3- and CD7-targeted LNPs to induce functional CAR T cells in vivo, resulting in efficient B cell depletion in preclinical models [307]. Notably, early-phase clinical trials are now exploring non-targeted LNP-based anti-TROP2 or anti-GPC3 CAR mRNA for treatment of solid tumors. Hunter et al. reported that CD8-targeted LNPs could be used to successfully deliver anti-CD19 CAR mRNA and generate CAR T cells in vivo, resulting in tumor control in humanized mice and B cell depletion in cynomolgus monkeys [308]. Meanwhile, CD8-targeted LNP CAR-T engineering is poised to enter clinical evaluation, with first-in-human trials anticipated in the near future [309]. Ongoing efforts aim to reprogram immune cells directly in vivo and to integrate CAR-T cell therapy with immune checkpoint inhibitors. These strategies may reduce CAR-T cell exhaustion and enhance therapeutic durability, collectively expanding the frontiers of cell therapy for cancer and autoimmune disorders.

Antibody-oligonucleotide conjugates (AOCs) are an emerging class of hybrid molecules that combine the targeting specificity of mAbs with the gene-modulating capacity of oligonucleotides. This class includs antisense oligonucleotides (ASOs), phosphorodiamidate morpholino oligomers (PMOs), small interfering RNAs (siRNAs), and aptamers [310]. The antibody component of an AOC provides cell- or tissue-specific delivery, whereas the oligonucleotide component modulates gene expression through exon skipping, RNA degradation, or mRNA knock-down. This design addresses one of the major limitations of RNA therapeutics, the difficulty of reaching extrahepatic tissues, particularly skeletal muscle or the central nervous system [310, 311]. For example, AOCs directed against TfR1 enhance uptake into muscle, brain, or tumor cells, thereby enabling modulation of disease-causing genes in target tissue [312].

Preclinical studies have highlighted the broad therapeutic potential of AOCs. In oncology, AOCs demonstrated efficacy in animal models by silencing oncogenes or enhancing tumor suppressor activity [313, 314]. In neuromuscular disorders such as Duchenne muscular dystrophy (DMD) and myotonic dystrophy type 1 (DM1), AOCs promote exon skipping or RNA interference, restoring dystrophin expression or reducing toxic RNA repeats in vivo [315]. Clinically, several AOC candidates are developing for rare diseases. For instance, AOC 1001, which targets TfR1 to deliver DMPK siRNA for the treatment of DM1, has progressed into phase III studies, demonstrating safety, target engagement, and preliminary efficacy in muscle RNA modulation (NCT07008469 and NCT06411288). Additional candidates from Dyne Therapeutics, including DYNE-101 for DM1 and DYNE-251 for DMD, are currently being evaluated in early-phase clinical trials and have received FDA Fast Track designations to expedite development [310]. For oncology indications, TAC-001 couples an anti-CD22 antibody with a TLR9 agonist and is under investigation in a phase I/II trial [316]. Drawing on the example of ADCs, continued progress in linker design, conjugation strategies, and supportive regulatory pathways is expected to facilitate the clinical approval of AOCs within the coming decade.

A major challenge in antibody-based cancer therapies is on-target, off-tumor toxicity, which compromises safety and limits efficacy. One promising solution to this issue is the implementation of activatable antibodies or antibody locks, which are engineered to remain inert in normal tissues but become selectively activated within the tumor microenvironment [317, 318]. These antibodies incorporate a masking peptide fused to the N-terminus of the light chain via a protease-cleavable spacer. In systemic circulation, the masking peptide blocks antigen recognition, minimizing on-target, off-tumor toxicity. However, tumor-associated proteases, such as matrix metalloproteinases (MMPs), matriptase, and urokinase-type plasminogen activator (uPA), will cleave the spacer and remove the masking domain to activate target binding. Thus, the antibody exerts therapeutic function only upon proteolytic activation in tumors, thereby broadening the therapeutic window [319].

This concept is broadly applicable to different antibody modalities, including ADCs, bsAbs and CAR-T therapies. A leading example of activatable antibody technology is the Probody platform [320]. Several Probody drug conjugates have entered clinical testing. Examples include the anti-CD166 ADC CX-2009, which is currently in phase I/II trials (NCT04596150); CX-2029, an anti-CD71 ADC in phase II evaluation (NCT03543813); and CX-2051, an anti-EpCAM ADC in a phase I study (NCT06265688). Together, these candidates highlight the promise of activatable antibodies to selectively expand the therapeutic window in oncology.

CX-2051 is a masked, conditionally activated Probody ADC that targets EpCAM, armed with a topoisomerase-1 inhibitor payload, designed to overcome safety challenges of EpCAM targeting by releasing its payload only in the tumor microenvironment. CX-2051 has demonstrated promising early clinical activity in heavily pretreated metastatic colorectal cancer. At the higher dose of 10 mg/kg, nearly 43% of evaluable patients experienced confirmed responses, significantly above the typical single-digit response rates seen in third-line CRC therapies. The median progression-free survival was 5.8 months, with most patients achieving disease control. Importantly, the safety profile was favorable, characterized by manageable adverse events and the absence of dose-limiting toxicities or severe pancreatic toxicity-addressing a major historical limitation associated with EpCAM-targeted therapeutics [321].

Looking ahead, the next wave of antibody innovation is expected to be driven by ML and AI. These approaches have already transformed the landscape of de novo antibody design, affinity maturation, and rational engineering. These data-driven approaches bridge computational prediction with experimental validation, offering new possibilities for designing antibodies with enhanced specificity, potency and developability. ML models trained on large-scale datasets, such as those derived from phage or yeast display, can reveal sequence-function relationships and predict antibody variants with improved binding affinity [48, 322, 323]. For instance, deep learning models trained on high-throughput FACS screening data have enabled the identification of high-affinity binders [324]. Additionally, pre-trained protein language models (PLMs), such as BERT-based architectures, can guide affinity optimization even in low-data contexts. For example, one recent study reported a 28.7-fold improvement in binding affinity by integrating PLM predictions with a yeast mating assay [46].

Deep generative models like RFdiffusion are capable of designing protein backbones and interfaces with high precision [52]. While initially successful in designing rigid binders, the challenge of accurately modeling flexible antibody loops, particularly the diverse CDR regions, has presented a critical barrier. To address this obstacle, Nobel laureate Dr. David Baker’s team developed a fine-tuned version of RFdiffusion which was used to create fully de novo VHHs and scFvs that bind specific disease-relevant epitopes; the design was performed entirely in silico [176]. Experimental validation using cryo-EM confirmed proper folding and epitope binding of designed antibodies against influenza hemagglutinin and *Clostridium difficile* toxin B. While these findings represent a significant step forward, the utilization of such methods remains limited to a few expert groups. Although ML and AI are poised to revolutionize antibody discovery and engineering, additional successful case studies and improvements in design accuracy will be crucial to realizing the full potential of AI-guided antibody design.

## Conclusions

Despite remarkable progress, antibody-based therapeutics continue to face challenges related to drug resistance, limited tissue penetration, and functional redundancy within signaling pathways. Advances in display technologies, structural epitope profiling, and biophysical optimization can facilitate the discovery of antibodies targeting functionally relevant epitopes. Nonetheless, monotherapies are often insufficient to cure disease. The development of bispecific or multi-specific antibodies, in combination with nanocarrier-mediated targeted delivery, offers a promising avenue to enhance efficacy and mitigate immune escape. In the future, the convergence of emerging modalities, such as antibody-conjugated LNPs, in vivo reprogramming of immune cells, and AI-driven antibody design, will likely redefine the development landscape for biologics. These platforms hold promise for more precise, scalable and patient-specific interventions. Furthermore, strategies to combine CAR-T cell therapy with immune checkpoint inhibitors and efforts to reduce CAR-T cell exhaustion are expanding the frontiers of cancer immunotherapy and autoimmune disease management. Beyond technological innovations, commercial strategies such as indication expansion and drug life-cycle management are increasingly recognized as essential components of therapeutic success. As the antibody field transitions toward a new era of programmable, multimodal, cross-disciplinary solutions, sustained progress will depend on the close integration of biological insights, engineering ingenuity, and strategic development frameworks.

## Future perspectives

Antibody-based therapeutics are poised to enter a new era of innovation, driven by advancements in antibody engineering, novel delivery technologies, and integrative platforms, such as AI. As the field continues to mature, next-generation modalities, streamlined development workflows, and expanding therapeutic applications are expected to redefine the clinical and commercial landscape of biologics.

### Emerging antibody modalities

The evolution from conventional mAbs to more complex, multifunctional modalities is a defining feature of the current and future therapeutic pipeline. Among new drugs, bsAbs represent a rapidly advancing class. By engaging two distinct antigens or epitopes, bsAbs enable novel mechanisms of action, such as immune cell redirection and dual pathway inhibition. The clinical utility of these technologies has been demonstrated by agents like blinatumomab and emicizumab; further, 178 bsAbs are currently in clinical development. Future directions include optimization of bsAb architectures to improve half-life, enhance tissue penetration, and reduce cytokine release syndrome.

ADCs are another expanding modality that leverages the specificity of antibodies to deliver potent cytotoxic payloads directly to target cells. Improved conjugation strategies, linker technologies and diversified payloads are anticipated to enhance the therapeutic indexes of novel ADCs. The recent successes of ADCs targeting HER2, TROP2 and BCMA reflect the potential of this platform in oncology and beyond.

BsAb-drug conjugates (bispecific ADCs) represent a novel class of therapeutics that integrate the dual-targeting capability of bsAbs with the cytotoxic potency of ADCs. Currently, several drugs in this class are in early-phase clinical development. These bispecific ADCs may overcome limitations of conventional ADCs associated with antigen heterogeneity and resistance. By simultaneously binding two distinct tumor-associated antigens or binding one tumor antigen and engaging T cells, bispecific ADCs have the potential to enhance tumor selectivity, improve internalization, and reduce off-target toxicity. Several bispecific ADCs are being evaluated in hematologic malignancies and solid tumors. Preclinical data demonstrate improved efficacy and broader therapeutic windows compared to monospecific ADCs. Key candidates include ADCs targeting HER2 × HER3 or HER2 × CD63, as these candidates show promise for treatment of tumors with low HER2 expression.

Looking ahead, the field is poised for rapid growth due to expected advancements in site-specific conjugation, linker-payload optimization, and tumor-selective bispecific formats. Challenges such as manufacturing complexity and immunogenicity remain, but progress in antibody engineering and bioinformatics-guided target selection is accelerating development. Ultimately, bispecific ADCs are expected to expand the treatable patient population and enable precision-targeted cytotoxic therapy across a wider range of cancers.

Nbs, or VHHs, offer benefits due to their small size, stability and superior tissue penetration. Clinical validation of Nb therapeutics, such as caplacizumab, underscores their utility in hematologic and autoimmune indications. Ongoing efforts to construct synthetic libraries and multivalent constructs may further expand their therapeutic relevance.

CAR-T therapy has transformed treatment of hematological malignancies. However, challenges remain in extending CAR-T cell efficacy to solid tumors. Expected innovations include switchable CAR designs, combinatorial antigen targeting, in vivo CAR-T therapy and enhanced trafficking mechanisms. These improvements are likely to enhance therapeutic outcomes and safety. The potential expansion of CAR-T therapy indications into autoimmune and infectious diseases is an area of growing interest.

### Artificial intelligence in antibody engineering

AI and ML technologies are increasingly being integrated into antibody discovery and optimization workflows. These approaches enable prediction of antigen-binding affinity, immunogenicity and paratope-epitope interactions, thus accelerating the design and engineering of therapeutic candidates. Deep learning models are capable of mining large-scale antibody libraries, identifying high-affinity clones, and improving stability profiles.

Notably, the development of structural prediction tools such as AlphaFold and Rosetta has significantly enhanced modeling of antibody-antigen complexes at high resolution. Use of such tools is expected to become standard in rational antibody design, facilitating in silico affinity maturation and structural optimization. Furthermore, the integration of AI in developability assessments and lead candidate selection is anticipated to reduce attrition rates and development timelines.

### mRNA-LNP-mediated in vivo antibody delivery

The success of mRNA-based vaccines has catalyzed interest in the use of mRNA-LNP platforms for in vivo expression of therapeutic antibodies. This approach enables transient antibody production directly in patients, obviating the need for recombinant protein manufacturing and potentially improving pharmacokinetics and tissue distribution.

Recent studies have demonstrated the feasibility of producing mRNA-encoded antibodies against viral targets such as SARS-CoV-2 and Ebola virus. Continued optimization of mRNA stability, translation efficiency and LNP composition is expected to improve therapeutic outcomes. This platform represents a particularly attractive strategy for rapid deployment of prophylaxis measures in infectious disease outbreaks, as well as for personalized cancer immunotherapies.

### Humanization and immunogenicity mitigation

Reducing immunogenicity remains a critical goal in the development of therapeutic antibodies. Traditional approaches such as chimerization and CDR grafting have been instrumental in this regard, while current efforts are increasingly focused on fully hAb formats derived from transgenic animal platforms (e.g., HuMab Mouse, VelocImmune) or synthetic libraries. These methods have led to the approval of several low-immunogenicity antibodies with extended half-life and improved tolerability.

Future strategies may involve deimmunization via T cell epitope elimination, AI-guided prediction of immunogenic hotspots, and individualized antibody design based on patient-specific HLA profiles. These approaches will be essential for developing drugs that can be used in chronic treatment settings and for patients with pre-existing anti-drug antibodies.

### Expanding therapeutic targets and indications

The therapeutic scope of mAbs is expanding rapidly beyond oncology and autoimmune diseases to encompass neurological, infectious, cardiovascular and metabolic disorders. Antibodies targeting amyloid-beta and tau have been evaluated for the treatment of Alzheimer’s disease, and recent regulatory approvals of antibody drugs signal a potential breakthrough in neurodegenerative disease therapeutics.

Antibody-based antivirals have also undergone rapid clinical translation, as exemplified by REGEN-COV and Inmazeb. Future directions include broad-spectrum antiviral antibodies, engineered Fc variants with enhanced effector functions, and platform technologies for pandemic preparedness. Antibodies against intracellular targets, previously considered inaccessible, are now being explored using cell-penetrating antibody formats and intrabody technologies.

### Fc engineering and functional modulation

Engineering of the Fc region continues to be a major focus for improving antibody pharmacodynamics and pharmacokinetics. Fc variants have been developed to prolong half-life (e.g., via enhanced FcRn binding), augment immune effector functions (e.g., ADCC and ADCP), or suppress undesirable immune activation. Fc-silent formats are also increasingly employed for non-cytotoxic indications, such as autoimmune and neurodegenerative diseases.

In the future, conditionally active Fc constructs that respond to environmental cues (e.g., pH, proteases) may enable spatial and temporal control of antibody activity. Additionally, bsAbs with engineered Fc regions may allow for tailored immune cell engagement in complex disease environments.

### Regulatory and manufacturing innovations

The COVID-19 pandemic revealed the feasibility of accelerated regulatory pathways for biologics. Harmonized global regulatory frameworks, real-time review models, and validated surrogate endpoints could further streamline antibody-based drug development and approval.

Animal-free approaches in preclinical and IND-enabling studies are emerging as viable alternatives to traditional in vivo models. This shift in approach is driven by ethical concerns, regulatory encouragement, and technological advances. Currently, in vitro assays using human-derived organoids, microphysiological systems (e.g., organ-on-a-chip), and 3D cell cultures are increasingly used to assess pharmacodynamics, toxicity, and efficacy. Computational modeling and in silico simulations further complement these platforms for pharmacokinetic and safety predictions.

Regulatory bodies like the FDA and EMA are showing increasing openness to these alternative analyses, especially when supported by a sound scientific justification. However, it is unlikely that full replacement of animal studies will occur in the near future, particularly for systemic toxicity and immunogenicity assessments.

Future work will focus on validating and standardizing these alternative methods to ensure regulatory acceptance. Integration of AI, multi-omics, and humanized in vitro models is expected to enhance predictive accuracy. As technology matures, animal-free platforms may become central to safer, faster, and more ethical antibody development pipelines.

Regarding manufacturing, advances in continuous bioprocessing, modular production platforms, and cell-free expression systems are expected to improve scalability and reduce production costs. These innovations combined with improved analytics and quality control systems may facilitate decentralized manufacturing and improve global access to biologics, particularly in low- and middle-income countries.

### Market trends and accessibility

The global market for therapeutic antibodies surpassed USD 267 billion in 2024 and continues to expand, driven by the clinical success of agents such as adalimumab, pembrolizumab, dupilumab and nivolumab. The rise of biosimilars will play an increasingly important role in improving accessibility and affordability. Regulatory convergence and advanced analytical tools have accelerated the approval of high-quality biosimilars, particularly for oncology and autoimmune indications. Future market growth is expected to be driven by diversification of therapeutic targets, adoption of next-generation antibody formats, and integration with digital health and companion diagnostics.

mAb therapeutics are entering a dynamic phase of development, marked by innovations across discovery platforms, therapeutic formats, delivery systems and regulatory paradigms. The further integration of AI, mRNA technology, and synthetic biology has also accelerated the translation of antibody candidates and enabled the design of multifunctional biologics with enhanced precision, efficacy and safety. As these trends converge, the mAb platform is anticipated to remain a cornerstone of modern medicine, giving rise to new therapies that are more personalized, precise, durable and accessible. Continued interdisciplinary collaborations and global investment will be critical to fully realize the therapeutic potential of antibodies in the coming decades.

## Data Availability

No datasets were generated or analysed during the current study.
